# Acute Versus Chronic Loss of Mammalian *Azi1/Cep131* Results in Distinct Ciliary Phenotypes

**DOI:** 10.1371/journal.pgen.1003928

**Published:** 2013-12-26

**Authors:** Emma A. Hall, Margaret Keighren, Matthew J. Ford, Tracey Davey, Andrew P. Jarman, Lee B. Smith, Ian J. Jackson, Pleasantine Mill

**Affiliations:** 1 MRC Human Genetics Unit, Institute of Genetics and Molecular Medicine at The University of Edinburgh, Western General Hospital, Edinburgh, United Kingdom; 2 Electron Microscopy Research Services, Medical School, Newcastle University, Newcastle, United Kingdom; 3 Centre for Integrative Physiology, School of Biomedical Sciences, University of Edinburgh, Edinburgh, United Kingdom; 4 MRC Centre for Reproductive Health, University of Edinburgh, The Queen's Medical Research Institute, Edinburgh, United Kingdom; Washington University School of Medicine, United States of America

## Abstract

Defects in cilium and centrosome function result in a spectrum of clinically-related disorders, known as ciliopathies. However, the complex molecular composition of these structures confounds functional dissection of what any individual gene product is doing under normal and disease conditions. As part of an siRNA screen for genes involved in mammalian ciliogenesis, we and others have identified the conserved centrosomal protein Azi1/Cep131 as required for cilia formation, supporting previous *Danio rerio* and *Drosophila melanogaster* mutant studies. Acute loss of *Azi1* by knock-down in mouse fibroblasts leads to a robust reduction in ciliogenesis, which we rescue by expressing siRNA-resistant *Azi1-GFP*. Localisation studies show Azi1 localises to centriolar satellites, and traffics along microtubules becoming enriched around the basal body. Azi1 also localises to the transition zone, a structure important for regulating traffic into the ciliary compartment. To study the requirement of *Azi1* during development and tissue homeostasis, *Azi1* null mice were generated *(Azi1^Gt/Gt^)*. Surprisingly, *Azi1^Gt/Gt^* MEFs have no discernible ciliary phenotype and moreover are resistant to *Azi1* siRNA knock-down, demonstrating that a compensation mechanism exists to allow ciliogenesis to proceed despite the lack of Azi1. Cilia throughout *Azi1* null mice are functionally normal, as embryonic patterning and adult homeostasis are grossly unaffected. However, in the highly specialised sperm flagella, the loss of Azi1 is not compensated, leading to striking microtubule-based trafficking defects in both the manchette and the flagella, resulting in male infertility. Our analysis of *Azi1* knock-down (acute loss) versus gene deletion (chronic loss) suggests that *Azi1* plays a conserved, but non-essential trafficking role in ciliogenesis. Importantly, our *in vivo* analysis reveals *Azi1* mediates novel trafficking functions necessary for flagellogenesis. Our study highlights the importance of both acute removal of a protein, in addition to mouse knock-out studies, when functionally characterising candidates for human disease.

## Author Summary

Cilia are slender projections from the surface of most mammalian cells and have sensory and sometimes motile functions. They are essential for mammalian development and defects in cilia lead to a group of human diseases, termed ciliopathies, with variable symptoms including embryonic lethality, lung and kidney defects, blindness and infertility. Cilia are complex structures composed of hundreds of components, whose individual functions are poorly understood. We screened for mammalian genes important in building cilia, and identified *Azi1/Cep131*, a gene previously shown to be required for cilia formation and function in fish and flies. We show that if we acutely reduce levels of Azi1 in mouse cells, fewer cells form cilia, but if we generate cells chronically lacking all Azi1, cilia form normally. In addition, mice without any Azi1 are healthy and viable, confirming normal cilia function. However, in these mice, the highly specialised ciliary structure of the sperm tail does not form, resulting in male infertility. We suggest Azi1 has conserved trafficking roles in both primary cilia and the specialised sperm flagella. Abruptly removing Azi1 results in instability causing the existing cilia network to collapse, whereas chronic deletion of Azi1 allows the system to re-equilibrate, and cilia to form normally.

## Introduction

Centrosomes are conserved animal organelles which function as the major microtubule organising centre (MTOC), and are required for diverse processes including formation of cilia and flagella, intracellular trafficking events, cell polarity and division. Structurally, the centrosome consists of a pair of cylindrical centrioles surrounded by a proteinaceous matrix of pericentriolar material (PCM) [1]. Importantly, centrioles replicate only once per cell cycle and are essential for the formation of cilia, key signalling organelles during development and homeostasis. In post-mitotic cells, the centrosome moves to the apical surface where the mother centriole docks with the cell membrane to become a basal body and a template for the axonemal microtubules of the primary cilium. Cilia assembly and function requires diverse trafficking events, including intraflagellar transport (IFT), coordinated by the basal body ‘hub’, which regulates traffic in and out of the ciliary compartment. Enrichment of key signalling receptors and downstream effectors in cilia allows these structures to function as effective signalling organelles, with unique protein and lipid composition [2]. The transition zone, a highly specialised structure just distal to the basal body, is thought to act as an additional ciliary gate, controlling traffic into and out of the cilium [3]. Many of these aspects of ciliogenesis are highly conserved [4].

Despite these common features, cilia are also structurally and functionally diverse. Cilia play important sensory roles, acting as transducers of developmental signalling pathways, detecting fluid flow, as well as highly specialised sensory receptors [5]. Some cilia are motile, involved in generating fluid flow in the embryonic node, airways, oviduct and brain, as well as in the propulsion of sperm. How the core ciliary assembly programme is modified and elaborated on to account for these species- and cell-specific variations is not well understood [4].

Mutations in conserved cilial and centrosomal genes have been identified in a growing spectrum of clinical disorders, termed ciliopathies, with both distinct and overlapping clinical features including polydactyly, skeletal defects, situs inversus, infertility and neuropathology [6], [7]. Proteomic and genetic studies in several organisms estimate the molecular composition of cilia/centrosomes to include hundreds to thousands of putative components, many of them unknown [8], [9]. Functional dissection of the role and requirement of many of these ciliopathy candidates in cilia formation and function are often performed using cell culture [10], [11], [12] and zebrafish knock-down models [13]. Mouse mutant models are analysed less often as these are more costly in time and resources to produce. Given the phenotypic complexities of clinical features in ciliopathies [14], what is the best way to understand the underlying molecular mechanisms for candidate genes in relation to human disease?

Recently, centriolar satellites have reported to be the site of localisation of many ciliopathy proteins, and are involved in their ciliary targeting, including OFD1 (oral-facia1-digital syndrome 1), BBS4 (Bardet-Biedl Syndrome 4) and CEP290 [15]. Conserved among vertebrates, but not present in arthropods, centriolar satellites are electron dense, multi-protein complexes enriched in the area surrounding the centrosome/basal body [16], [17], [18]. These are dynamic structures trafficking along microtubules towards the centrosome utilising dynein motors [16], [18]. Centriolar satellites have been shown to regulate ciliogenesis and centriole biogenesis, in part by regulating trafficking of proteins to and/or sequestering of proteins away from the centrosome/basal body [19], [20], [21], [22]. Centriolar satellites are defined by pericentriolar material 1 (PCM1) which is a key scaffolding component of centriolar satellites and to date, all centriolar satellite-localised proteins have been shown to interact with PCM1 [17]. However, what the functional significance of vertebrate-specific centriolar satellites to mammalian development is and how they affect the function of highly conserved components in ciliogenesis and centriole biogenesis is unknown.

Here, we address the role and requirement of 5-*az*acytidine *i*nduced gene *1* (Azi1)/Cep131 (MGI:107440), a highly conserved centrosomal protein, in ciliogenesis. We screened a subset of cilia-enriched orthologous candidates from *Drosophila melanogaster* studies [23] by RNAi to identify genes involved in mammalian ciliogenesis, and identified *Azi1/Cep131*. This finding agrees with a previous siRNA screen, which showed a role for the human orthologue, *AZI1*, in ciliogenesis [10]. Both *Drosophila melanogaster* mutants and *Danio rerio* morphants of *Azi1* (*dila/CG1625* and *cep131*, respectively) phenocopy mutations of known ciliary genes [24], [25], suggesting Azi1 plays a conserved function in ciliogenesis. AZI1 was described as a centrosomal protein (*Ce*ntrosomal *p*rotein *131*: Cep131) in a large scale proteomics screen and this localisation was recently refined to the centriolar satellites [26], [27], [28]. The mouse protein is highly expressed in the testes in germ cells during the period of flagellar formation [29]. More recently, additional roles for AZI1 include involvement in genome stability and centriole duplication. Knock-down of *AZI1* leads to an increase in double-stranded DNA breaks, indicated by γH2AX staining, as well as a slight increase in cells with extra centrioles [28], [30]. However, little is known of the *in vivo* role of mouse Azi1 and its requirement for development.

Here we utilise knock-down, localisation and live-imaging techniques, to further investigate the role of Azi1 in mammalian ciliogenesis at the cellular level. To determine the requirement for *Azi1* in mouse development, we generated *Azi1* null mutant mice and focused on the *in vivo* role of Azi1 in ciliogenesis and genome stability. Our analysis of *Azi1* knock-down (acute loss) versus gene deletion (chronic loss) suggests that *Azi1* plays a conserved, but non-critical trafficking role in ciliogenesis. Importantly, our *in vivo* analysis reveals *Azi1* mediates novel trafficking events necessary for spermiogenesis and male fertility.

## Results

### Functional cell-based screening of putative ciliary candidates from *Drosophila* identifies *Dila* orthologue *Azi1* as required for mammalian ciliogenesis

Using a set of forty orthologous putative ciliary genes identified as highly expressed in ciliated cell types in *Drosophila melanogaster* [23], we carried out an siRNA screen in a mouse fibroblast cell line to identify genes involved in mammalian ciliogenesis. Cilia formation was assayed as the percentage of cells with a cilium, marked by anti-Arl13b, a ciliary membrane marker [31], and anti-acetylated α-Tubulin, a ciliary axoneme marker, using an automated imaging and image analysis system.

**Figure 1:**
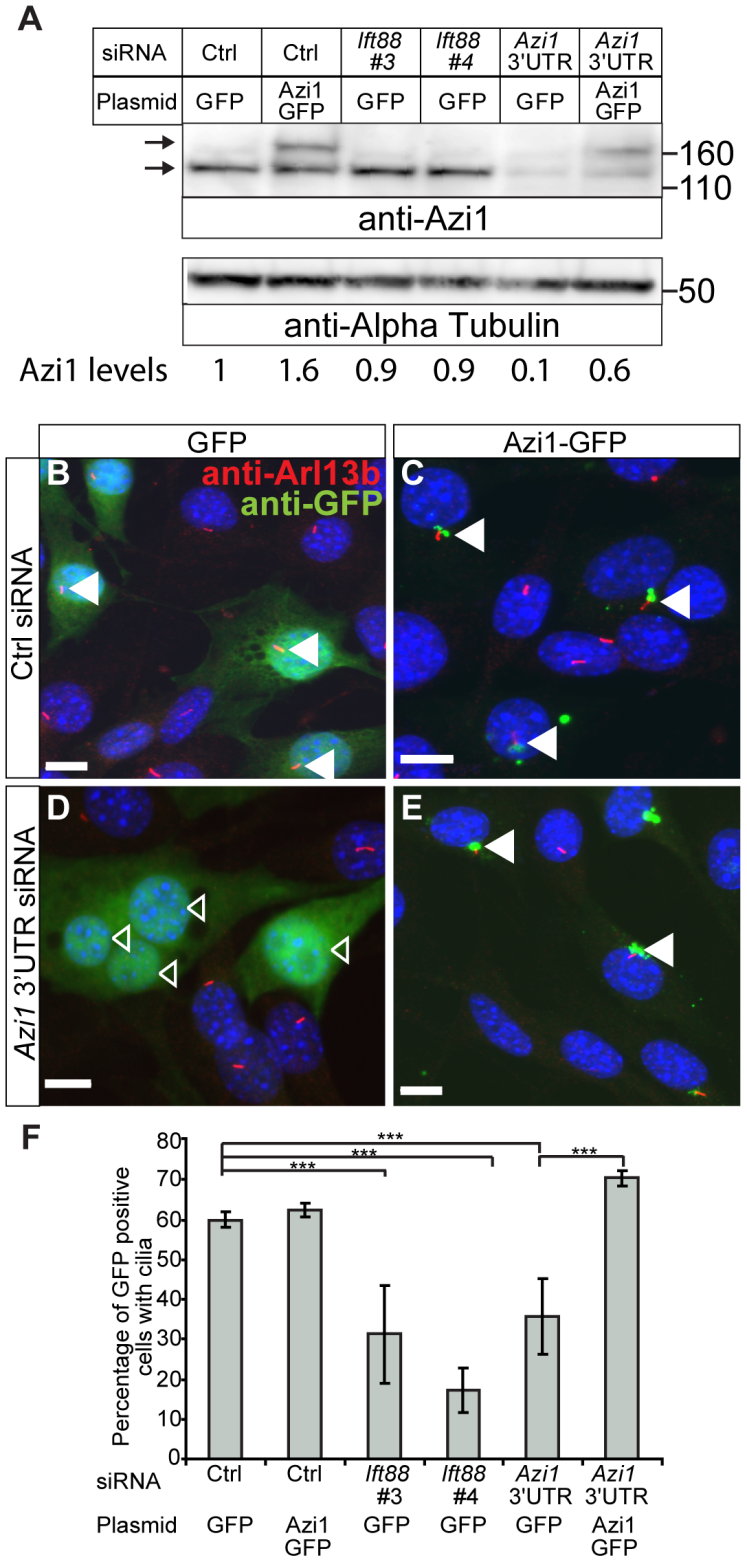
*Azi1* knock-down leads to reduced ciliogenesis. Mouse ShhLIGHT-II fibroblast cells were transfected with siRNA (a non-targeting control siRNA (Ctrl), two positive control siRNAs targeting *Ift88* (Ift88 #3 and #4) or a pool of four siRNAs targeting the 3′ UTR of *Azi1* (*Azi1* 3′UTR)), along with plasmids encoding either *GFP* or *Azi1-GFP* (which lacks the 3′UTR of *Azi1*). (A) Western blot probed with anti-Azi1 antibody (SF91) shows endogenous Azi1 levels are reduced upon *Azi1* 3′UTR siRNA addition (lower arrow). Tagged Azi1-GFP protein is also detected (upper arrow). The blot was reprobed with anti-α Tubulin as a loading control. Below is quantification of total Azi1 levels (endogenous plus overexpressed), relative to α-Tubulin. (B–E) Cells were stained with anti-Arl13b to mark the cilia and GFP Booster (Chromotek) to enhance the GFP signal. Transfected cells are highlighted with arrowheads; closed arrowheads highlight cells with cilia, open arrowheads highlight cells without cilia. (F) Addition of *Azi1* 3′UTR siRNA significantly reduced the percentage of cells with cilia, and this reduction is rescued to wild type levels by co-transfection with *Azi1-GFP*. Shown is the mean +/− SEM of two technical and two biological duplicates (*** *P*<0.001, chi-squared test). Scale bars represent 10 µm.

We identified *Azi1/Cep131* as the top hit for genes involved in cilia formation, with at least two of four siRNAs giving a significant reduction in ciliogenesis across three independent assays (data not shown). This observation is supported by the study of Graser *et al.* (2007), who found a reduction in ciliogenesis on *AZI1* knock-down in human hTERT-RPE1 cells [10]. To exclude off-target effects of the siRNAs, we co-transfected a different pool of four siRNAs, specifically targeting the 3′ untranslated region (UTR) of only *Azi1*, along with a plasmid encoding either *GFP* or *Azi1-GFP.* The *Azi1-GFP* plasmid lacks the 3′ UTR of *Azi1* and so is resistant to these siRNA. Transfection of *Azi1* 3′ UTR siRNA leads to a reduction in Azi1 protein to 10% of wild type levels, which can be partially rescued by co-transfection with *Azi1-GFP* (Figure 1A). *Azi1* knock-down leads to a 50% reduction of transfected cells with cilia (Figure 1B, D and F), similar to that seen with a positive control siRNA targeting *Ift88*, a gene essential for ciliogenesis [32], [33]. Importantly, co-expression of *Azi1-GFP* rescues the phenotype back to control levels demonstrating that the ciliary phenotype observed upon addition of *Azi1* siRNA is not due to off-target effects of the siRNA (Figure 1C, E and F). We conclude that *Azi1* is involved in mammalian cilia formation.

### Azi1 traffics along microtubules towards the centrosome/ciliary base, where it localises to the transition zone

**Figure 2:**
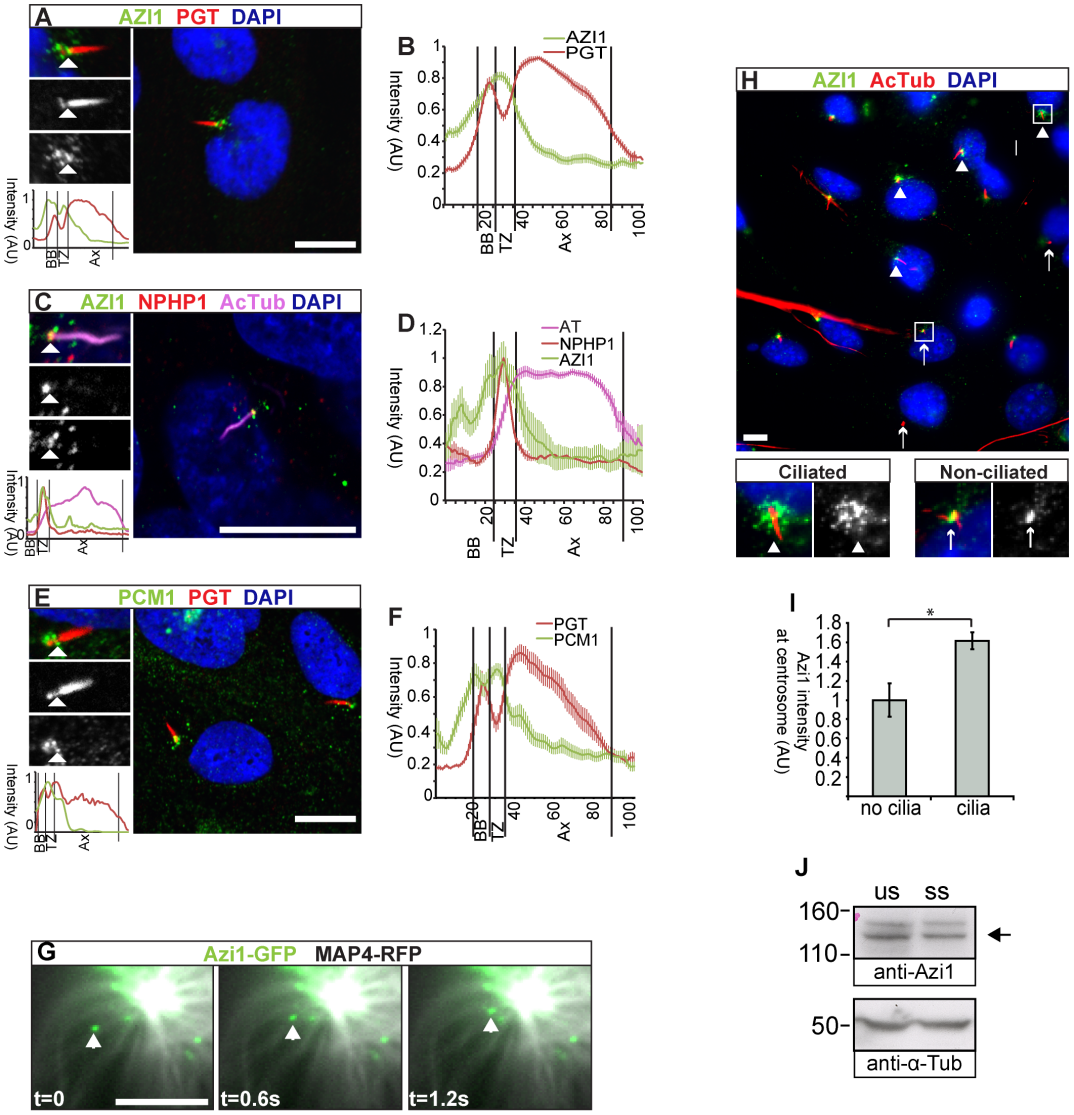
Azi1 localises to centriolar satellites and the transition zone, and traffics along microtubules. (A–F) hTERT-RPE1 cells, (A) stained with anti-polyglutamylated tubulin (PGT), marking the cilium and basal body, but absent from the transition zone and anti-AZI1 (Abcam; ab110018). AZI1 localises to the transition zone (TZ). (B) Average intensity profiling confirms localisation of AZI1 at the TZ (n = 30). (C) AZI1 localisation at the TZ was further confirmed by co-staining with anti-NPHP1, which marks the TZ and anti-acetylated α-Tubulin (AcTub) marking the axoneme. (D) Average intensity profiles show AZI1 colocalises with NPHP1 (n = 10). (E) Centriolar satellite marker PCM1 also localises to the TZ, identified by the absence of anti-PGT staining between the axoneme and the basal body. (F) Average intensity profiling shows PCM1 localisation to the TZ (n = 12). (A, C, E) Enlargements highlight the cilium and show separate channels. Arrowheads highlight the TZ. Below is an intensity graph, highlighting the enrichment of AZI1 (A, C) or PCM1 (E) at the transition zone. (B, D, F) Plotted is mean +/− SEM. BB: basal body, Ax: axoneme, AU: arbitrary units. (G) Azi1-GFP (green) traffics along microtubules marked by Map4-RFP (grayscale) in NIH-3T3 cells. Images were taken at 600 ms intervals (t: time, s: seconds). (H) Anti-acetylated α-Tubulin marks the ciliary axoneme, basal bodies and the centrosomes of unciliated cells. Anti-Azi1 staining is more intense at the basal body in ciliated cells (arrowheads) than the centrosome of unciliated cells (arrows). Mean intensity of pericentrosomal Azi1 staining is quantified in (I). * *P*<0.05, Student's t-test, n = 139 cells, two independent experiments. (J) AZI1 protein levels (anti-Azi1 SF91) in unsynchronised (us) or serum starved (ss) hTERT-RPE cells. Levels of Azi1 (arrow) remain unchanged when cells are serum starved to induce ciliogenesis. A presumed non-specific band at 150 kDa also detected by anti-Azi1 SF91, is more prominent in human cells. Scale bars represent 10 µm (A, C, E and H) or 1 µm (G).

AZI1 was originally identified as a **ce**ntrosomal **p**rotein (Cep131 [26]). We have spatially refined the localisation of mouse endogenous and GFP-tagged Azi1 to centriolar satellites, marked by anti-PCM1, which confirms recent human AZI1 immunofluorescence reports (Figure S1A and G) [28], [34]. We identified a further pool of human and mouse Azi1 at the transition zone (Figures 2A, B and S1B–C), indicated by co-staining with anti-polyglutamylated tubulin, which stains the ciliary axoneme and basal body but, importantly, is absent from the transition zone [35]. The transition zone is an area at the base of the cilia involved in regulating traffic into the cilium [3]. Co-staining with anti-NPHP1, a marker of the transition zone [36], [37], confirms this localisation (Figures 2C, D and S1D). This is consistent with the observation that in *D. melanogaster* ciliated sensory neurons *Azi1* homologue *dila* localises distal to the basal body at the putative transition zone [24].

Recently, CEP290 has also been reported to localise to both centriolar satellites and the transition zone [38], [39], [40], raising the possibility this could be a general trend for centriolar satellite proteins. To test this, we investigated the localisation of PCM1, the core component of centriolar satellites at the transition zone. Indeed, PCM1 localises to the transition zone of most, but not all cilia, as indicated by the gap between the basal body and axoneme on anti-polyglutamylated tubulin staining (Figure 2E and F). Previous reports had shown OFD1 and PCM1 to similarly localise to the distal portion of basal bodies [15]. Interestingly, the putative functional orthologue of OFD1, UNC, is also found at the putative transition zone of *Drosophila* mechanosensory neurons [24], [41]. Together, this suggests that docking at the transition zone may be a conserved feature of components of mammalian centriolar satellites.

We used live imaging of Azi1-GFP to address the dynamics of Azi1 localisation. In interphase cells, centriolar satellites have been proposed to function in dynein motor-dependent, microtubule-based trafficking of proteins to the centrosome [19], [40], and it has been shown recently that the pericentriolar localisation of AZI1 is microtubule dependent [28]. Similarly, trafficking of cargo associated with IFT motors along microtubules into the ciliary compartment is selectively regulated in part by the transition zone [42]. To examine Azi1 trafficking more directly we imaged Azi1-GFP movement in live mouse NIH-3T3 cells. Azi1-GFP was observed to traffic along microtubules, co-labelled with Map4-RFP (Figure 2G). Azi1-GFP traffics with a dynamic saltatory motion, with periods of fast movement, for an average distance of 3.4 µm (range 1.3–10.2 µm) interspersed with sometimes long stationary periods. Azi1-GFP was observed to move both towards and away from the centrosome, similar to observations of PCM1-GFP [18] (Movie S1). Although average speeds varied according to direction, 1.8±0.2 µm/s (mean ± SEM) towards the minus end of microtubules at the centrosome, and 1.0±0.3 µm/s away from the centrosome, they were consistent with speeds observed previously for microtubule-based motors *in vivo* [43], [44]. This suggests Azi1 is involved in microtubule-based trafficking to and from the centrosome/basal body.

Higher levels of endogenous AZI1 staining are observed around basal bodies and surrounding centriolar satellites of ciliated cells compared to non-ciliated cells (Figure 2H and I). However, total levels of AZI1 do not change upon serum starvation (Figure 2J) indicating that under these conditions to induce ciliogenesis, there is a redistribution of AZI1 towards the basal body area.

It has been proposed that centriolar satellites act as proteinaceous scaffolds to physically restrict access of proteins to the centrosome/cilium complex [22]. Disruption of core components results in dissolution or dispersal of centriolar satellites, and relocalisation of associated centriolar satellite proteins to the centrosome/basal body [15], [20], [22], [40]. Despite its localisation, *Azi1* siRNA knock-down in mouse cells does not alter centriolar satellite integrity as shown by Pcm1 localisation, consistent with recent reports for human AZI1 [28]. This suggests Azi1 is not required for mammalian centriolar satellite integrity nor retention of Pcm1 to these structures (Figure S1E–H).

### *Azi1* is dispensable for mouse embryonic development

**Figure 3:**
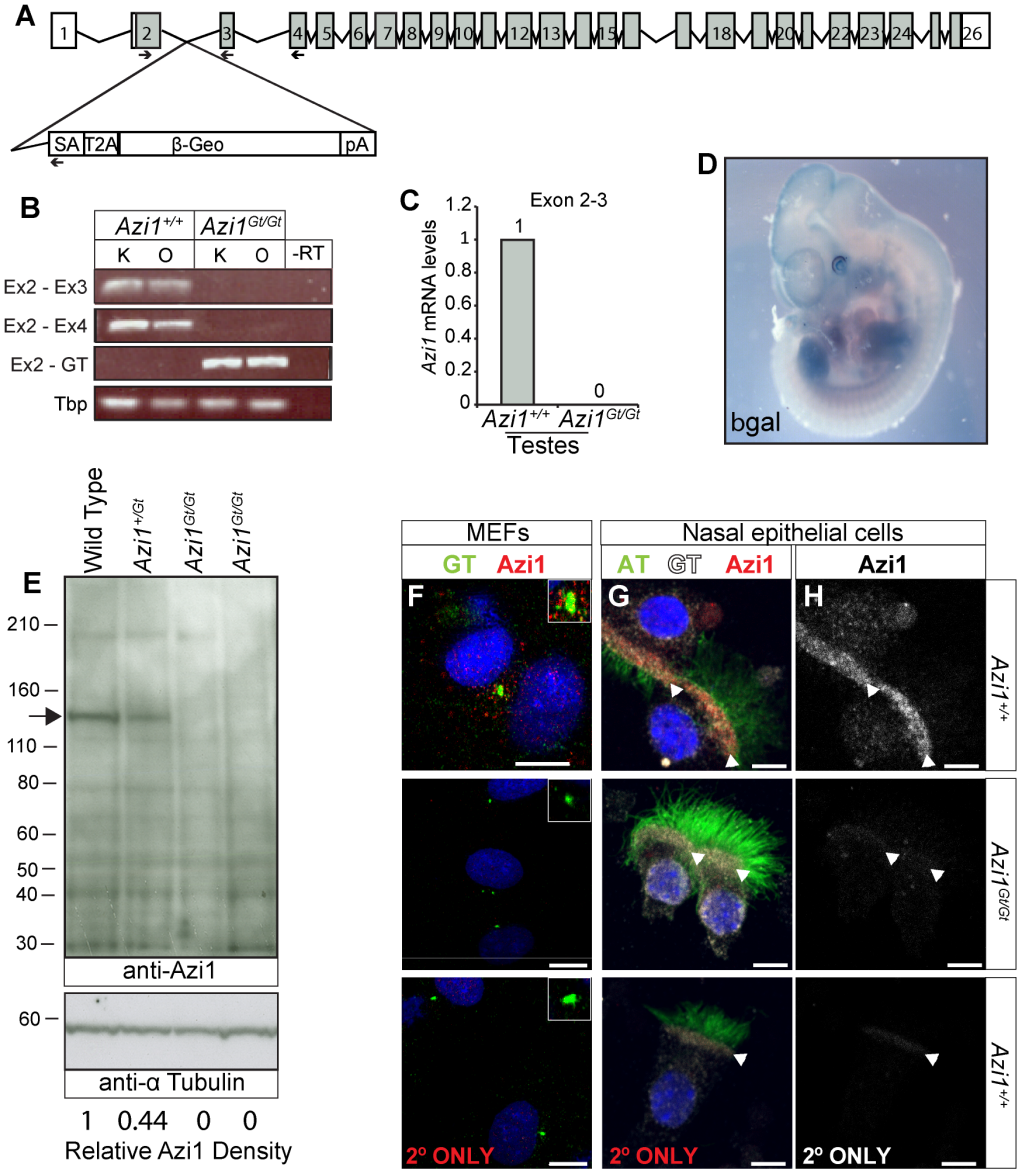
*Azi1^Gt^* is a null allele of *Azi1*. (A) Schematic of the *Azi1* gene trap inserted in intron 2 of *Azi1*. Exons are shown as boxes with translated transcript shaded. Arrows indicate primers used to characterise mRNA expression in *Azi1^Gt/Gt^* mice. SA: Splice acceptor, T2A: self-cleaving peptide, pA: polyA. (B) *Azi1* mRNA expression in kidneys (K) and ovaries (O) from *Azi1^+/+^* and *Azi1^Gt/Gt^* mice was examined. No expression was detected across the gene trap insertion site in *Azi1^Gt/Gt^* mice. (C) The lack of wild type transcription across the gene trap insertion site was confirmed by qPCR on testes cDNA. (D) LacZ staining of E11.5 *Azi1^Gt/+^* embryos demonstrates the gene trap-βGeo cassette is expressed. *Azi1* expression is ubiquitous during development with higher expression in the limbs, somite derivatives, eyes and brain. (E) No full length or truncated Azi1 protein expression was detected in *Azi1^Gt/Gt^* mutant testes. A strong band was detected in wild type and at lower levels in *Azi1^+/Gt^* testes extract using an antibody raised to the C-terminal of Azi1 (SF91) (See Figure S3A). Densitometry was used to give Azi1 levels relative to α-Tubulin, normalised to 1 in wild type (lower of panel D). *Azi1^Gt/Gt^* mice are null for *Azi1*. (F–H) Endogenous Azi1 is detected around centrosomes of wild type MEFs (F) and multiciliated airway epithelial cells (G and H). No Azi1 expression was detected by immunofluorescent staining of MEFs or nasal brush biopsies from *Azi1^Gt/Gt^* mice, further confirming these mice are null for Azi1. Bottom panel shows the secondary only control. Inset in F highlights the centrosome. Arrowheads in G and H highlight the apical surface where γ-Tubulin positive centrosomes dock, and where Azi1 is localised in wild type cells. Scale bars represent 10 µm (F–H).

Given the high conservation of *Azi1* among ciliates (Table S1), including arthropods which lack centriolar satellites, together with phenotypic mutant data from diverse organisms [24], [25], [45], [46], we predicted *Azi1* would have a central role in mammalian cilia biology *in vivo* and thus generated mouse mutants null for *Azi1. Azi1^Gt(CCOG35)Wtsi^* embryonic stem (ES) cells, which have a gene trap inserted into intron 2 of *Azi1* (Figure 3A), were used to generate *Azi1^+/Gt(CCOG35)Wtsi^* mice (referred to as *Azi1^Gt/+^* throughout this manuscript). *Azi1^Gt/Gt^* mice are born at sub-Mendelian ratios, with approximately two thirds of the expected numbers of *Azi1^Gt/Gt^* mice remaining after weaning (see Table 1, *P* = 0.0025). A significant reduction in *Azi1^Gt/Gt^* numbers was observed at embryonic day 11.5–13.5 (E11.5–13.5), suggesting roughly a third of mutants are lost before mid-gestation, although further work is needed to determine exactly when this loss occurs (Table 1). *Azi1^Gt/Gt^* mice that are born appear morphologically normal, and are the same weight as wild type littermates (Figure S5G and H). Viable *Azi1* mutant mice showed none of the gross abnormalities associated with cilia dysfunction in mice, including failure to thrive, hydrocephalus, situs inversus, and chronic airway infections.

**Table 1:** Genotypes of animals born from *Azi1^+/Gt^* x *Azi1^+/Gt^* matings.

Genotype	*Azi1^+/+^*	*Azi1^+/Gt^*	*Azi1^Gt/Gt^*	No. litters	Chi squared
**After weaning**	95	171	54	41	P = 0.0025
**E11.5–13.5**	26	61	15	14	P = 0.043

Azi1 has several coiled-coil domains as well as a predicted t-SNARE domain (IPR010989) implicated in membrane fusion events during vesicular transport (Figure S2B). The gene trap is predicted to truncate Azi1 after the initial 69 amino acids such that any remaining Azi1 trapped protein in *Azi1^Gt/Gt^* mice will lack all the predicted domains in the more highly conserved C terminus (Figure S2, Table S1), and hence unlikely to be functional. To confirm that the gene trap eliminated gene expression, we examined *Azi1* mRNA expression levels across the gene trap insertion site (Figure 3B and C). No expression across the insertion site was detected in kidneys, ovaries or testes from *Azi1^Gt/Gt^* mice by RT-PCR or qRT-PCR (Figure 3B and C), whereas robust expression of the gene trap was detected (Figure 3B). X-Gal staining of E11.5 *Azi1^Gt/+^* embryos further confirmed expression of the gene trap β-geo gene, and showed *Azi1* expression is ubiquitous during development, with higher expression in tissues with high levels of cilia-dependent developmental signalling such as the limbs, eyes, somite derivatives and brain (Figure 3D).

We detected some low level expression of the 3′ end of *Azi1* in *Azi1^Gt/Gt^* mice; when quantified by qRT-PCR this was less than 2% of wild type levels (Figure S2C–E). Importantly, no Azi1 protein was detected in *Azi1^Gt/Gt^* mice when probing with an antibody raised against the C terminal of Azi1 (Figure 3E and S2B), despite a single strong band of the expected size in *Azi1^+/+^* and a band of roughly 50% intensity in *Azi1^Gt/+^* samples. Furthermore, anti-Azi1 immunofluorescence analysis of *Azi1^Gt/Gt^* mutant mouse embryonic fibroblasts (MEFs) or multiciliated airway epithelial cells detected no signal, despite clear centrosomal/basal body localisation of Azi1 in littermate controls (Figure 3F–H). This suggests that any low level transcription detected at the 3′ end of *Azi1* in *Azi1^Gt/Gt^* mice is untranslated; indeed there are several predicted untranslated transcripts at the 3′ end of *Azi1* (ENSMUST00000156075, ENSMUST00000150463, ENSMUST00000144128 and ENSMUST00000145641). We conclude that *Azi1^Gt/Gt^* is a null allele of *Azi1*.

### Cilia, centrioles and centriolar satellites of *Azi1* null MEFs are grossly normal

**Figure 4:**
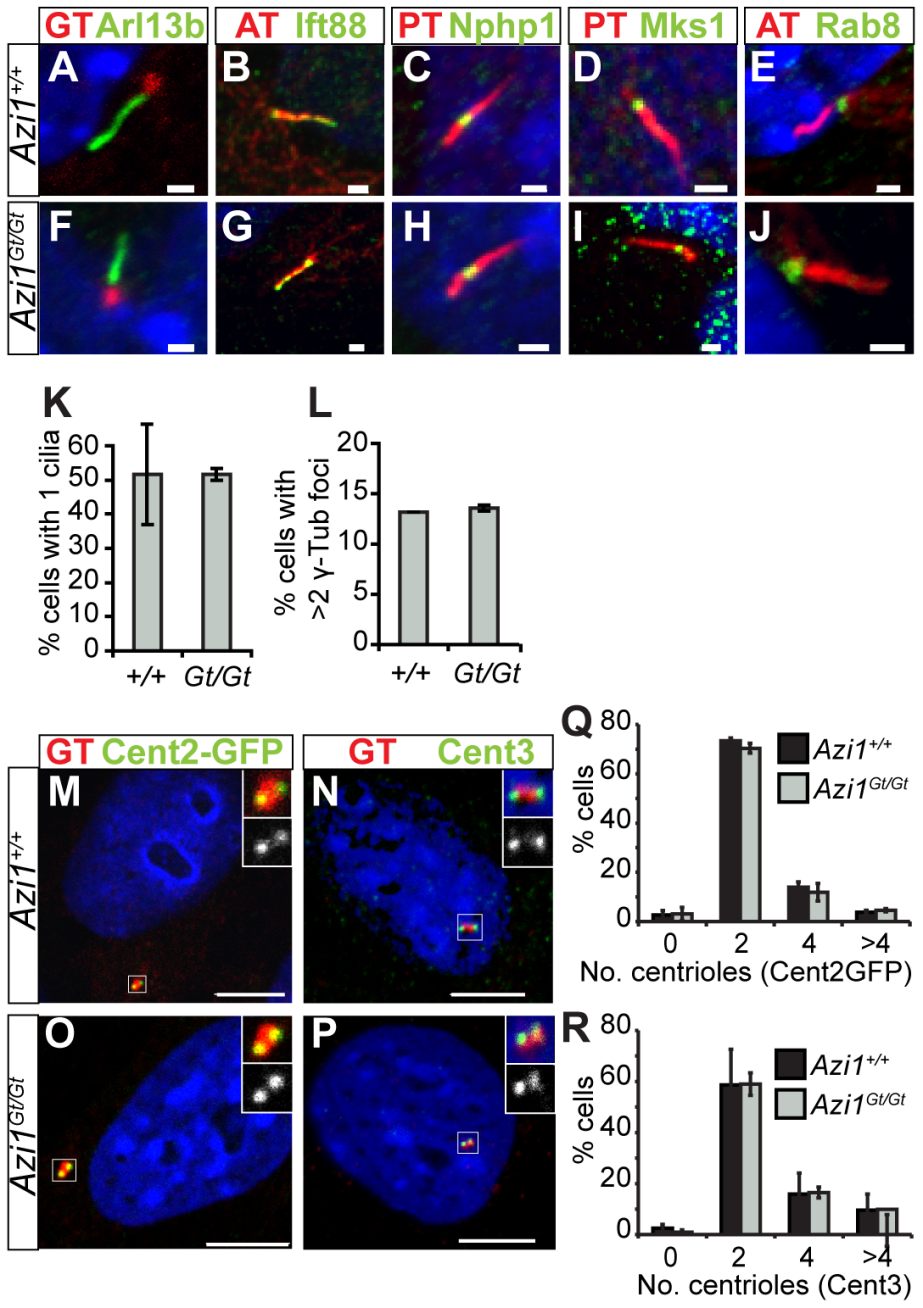
*Azi1^Gt/Gt^* MEF have normal cilia and centriole numbers. *Azi1^Gt/Gt^* MEFs have normal numbers of cilia, centrosomes and centrioles. *Azi1^Gt/Gt^* MEFs have normal cilia compartmentalisation as shown by localisation of ciliary membrane protein Arl13b (A and F), IFT-B protein Ift88 (B and G), transition zone markers Nphp1 and Mks1 (C, D, H and I) as well as normal localisation of small GTPase Rab8, involved in trafficking to the cilium (E and J) (GT: anti-γ-Tubulin, AT: anti-acetylated α-Tubulin, PGT: anti-polyglutamylated Tubulin). (K) Quantification of percentage of cells with a cilium, stained with anti-Arl13b (A and F), showing no difference between *Azi1^+/+^* and *Azi1^Gt/Gt^* MEFs. (L) Quantification of the number of cells with >2 centrosomes stained by anti-γ-Tubulin (A and F) again showing no difference between *Azi1^+/+^* and *Azi1^Gt/Gt^* MEFs. (M–R) *Azi1^Gt/Gt^* MEFs have normal numbers of centrioles, marked with either Centrin2-GFP (M and O) or anti-Centrin 3 (N and P), quantified in (Q) and (R), respectively. (K, L, Q and R) Shown is the mean +/−standard deviation, n = 3 cell lines, passage number <7. Scale bars represent 1 µm (A–J) or 10 µm (M–P).

Primary cilia are required for mammalian development, so the fact most *Azi1* null mice survive without any patterning defects suggests *Azi1* is not required for mammalian ciliogenesis *in vivo*. In contrast, transient siRNA knock-down of *Azi1* leads to a two-fold reduction in ciliogenesis (Figure 1). On careful examination of cilia formation in *Azi1* null primary MEFs we found normal numbers of cilia, marked by anti-Arl13b (Figure 4A, F and K). Moreover, cilia compartmentalisation appeared normal, with correct distributions of the ciliary membrane protein Arl13b, the IFT-B protein Ift88, and components of the transition zone Nphp1 and Mks1 (Figure 4A–D, F–I). Post-translational modifications of tubulin also appeared normal: acetylated α-Tubulin is present at the basal body and axoneme, polyglutamylated tubulin is present at the axoneme and basal body but absent from the transition zone, and γ-Tubulin is present at the basal body (Figure 4A–J). Localisation of Rab8, a small GTPase involved in trafficking to the cilium [21], whose localisation has been shown to be centriolar satellite-dependent [40], was also localised normally in *Azi1^Gt/Gt^* MEFs (Figure 4E and J). Together, this analysis suggests that *Azi1* is dispensable for primary cilia formation and compartmentalisation both *in vivo* and in primary cells.

Given its enrichment at centriolar satellites in mouse and human cells, where Azi1 interacts with Pcm1 (Figure S1) [28], [34], we examined formation of centriolar satellites in the absence of *Azi1*. Similar to *Azi1* knock-down, Pcm1 is correctly localised in *Azi1^Gt/Gt^* MEFs (Figure S3A–C), as are centriolar satellite components Cep72 and Cep290, involved in modulating localisation and composition of centriolar satellites [22], [40] (data not shown). This confirms Azi1 is not required for centriolar satellite formation, or localisation of key structural components like Pcm1, nor regulatory components like Cep290 or Cep72.

As centriolar satellites are proposed to have a role in regulating centrosome and centriole biogenesis, and *AZI1* knock-down in human cells leads to increased Centrin2-positive foci [17], [19], [28], [47], we examined the numbers of centrioles (marked by Centrin2-GFP and anti-Centrin3) and centrosomes (marked by anti-γ Tubulin) in cells lacking *Azi1* (Figure 4A, F, L–R). We found both centrosome and centriole numbers were normal in *Azi1^Gt/Gt^* MEFs (Figure 4A, F and N). Multiciliated cells have the ability to assemble hundreds of centrioles through two parallel pathways [48], [49], in both of which, protein-rich fibrous granules, akin to centriolar satellites, marked by PCM1, are found surrounding the elongating centrioles [18]. After assembly in the cytoplasm, these centrioles move apically to dock at the plasma membrane and each extend a ciliary axoneme. Ultrastructural analysis of motile multiciliated epithelia of adult trachea revealed normal numbers and docking of basal bodies and appendage formation in *Azi1* null mice (Figure S3D and G). Confirming the immunofluorescent analysis in *Azi1* mutant cells, transition zone ultrastructure appeared morphologically normal in *Azi1* null mice (Figure S3E and H).

In summary, and in contrast with the acute *Azi1* knock-down in both human and mouse cells (Figure 1) [10], [28], cilia and centriole structure appears grossly normal in *Azi1* null cells.

### *Azi1* null MEFs have compensated for the loss of Azi1

**Figure 5:**
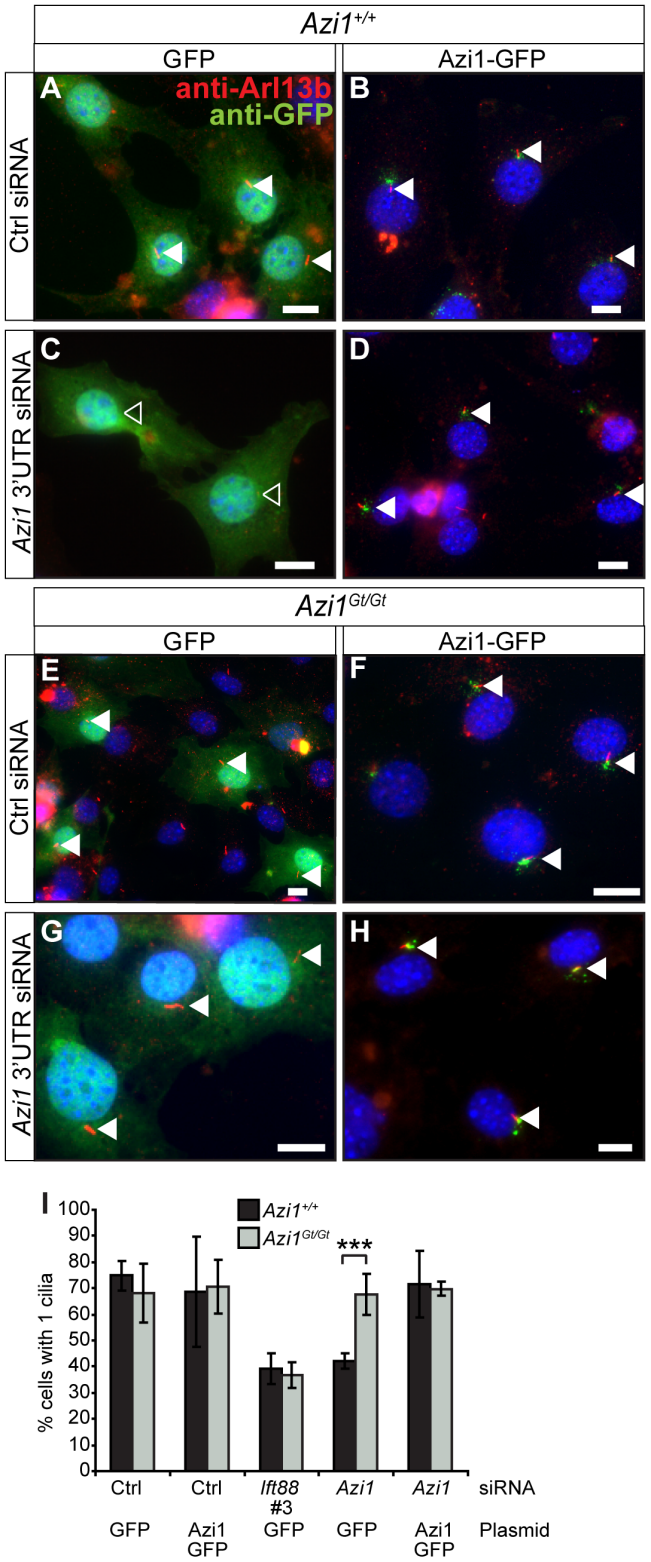
*Azi1* null MEFs have compensated for the loss of *Azi1*. *Azi1^+/+^* and *Azi1^Gt/Gt^* MEFs were co-transfected with siRNAs to *Ift88*, the 3′ UTR of *Azi1*, or a non-targeting control (Ctrl), along with plasmids encoding either *GFP* or *Azi1-GFP*. (A–H) Cilia are marked by anti-Arl13b (red), and anti-GFP Booster (green: Chromotek), in wild type (A–D) and *Azi1* null MEFs (E–H). (I) Quantification of the percentage of transfected cells with cilia. Wild type MEFs show a reduction in the percentage of cells with cilia upon treatment with *Azi1* siRNA, which is rescued by overexpressing Azi1-GFP. *Azi1^Gt/Gt^* MEFs show normal cilia numbers when transfected with non-targeting siRNA, and cilia numbers remain unchanged upon treatment with *Azi1* siRNA in *Azi1^Gt/Gt^* MEFs. Shown is mean +/− standard deviation (n = 3).

The difference in ciliary phenotypes observed with acute *Azi1* knock-down versus its chronic absence in *Azi1^Gt/Gt^* MEFs is intriguing. To rule out differences between the cell line used for the screen and primary cells, we co-transfected wild-type MEFs with siRNA against *Azi1* along with plasmids encoding either *GFP* or *Azi1-GFP* and examined cilia formation. Similar to the results obtained in the embryonic fibroblast cell line (Figure 1), *Azi1* knock-down in primary MEFs led to a robust reduction in ciliogenesis which was rescued by the siRNA-resistant Azi1-GFP (Figure 5A–D and I). To rule out any residual *Azi1* function in our mutant *Azi1^Gt/Gt^* cells, and to further eliminate off-target effects, we transfected *Azi1^Gt/Gt^* MEFs with *Azi1* siRNA. While our positive control *Ift88* siRNA gave a robust reduction in ciliogenesis in *Azi1^Gt/Gt^* MEFs, ciliogenesis was unaffected upon *Azi1* knock-down (Figure 5E–I), demonstrating that *Azi1* null MEFs have compensated for the loss of *Azi1*. Discrepancies between the phenotypic severity observed with siRNA knock-down versus genetic deletion has previously been attributed to the acute nature of knock-down, allowing less time for compensation to occur [50], [51]. We conclude that although *Azi1* is involved in cilia formation in mouse, compensation during embryogenesis in the absence of *Azi1* allows ciliogenesis to proceed normally in most tissues of *Azi1* null mice.

### A global DNA damage response is not observed in *Azi1* null mice

A role for cilia/centrosomal proteins in genome stability and DNA damage response pathways has been proposed [52], and was recently reported for *AZI1* [28], [30]. Unlike the reported *AZI1* knock-down [28], [30], we saw no increase in the number or intensity of γH2AX foci in *Azi1* null MEFs (Figure S4A, F and K and data not shown). To test whether *Azi1* mutants were more susceptible to DNA damaging agents, we challenged MEFs with either hydroxyurea (HU) or ionising radiation and examined γH2AX foci. No significant difference was observed between genotypes at lower concentrations of HU or upon challenge with ionising radiation, although *Azi1* null MEFs were more susceptible to high doses of HU (5 mM) (Figure S4B–E, and G–K). As Staples *et al.* (2012) reported an increase in micronuclei in *AZI1* depleted U2OS cells [28], we examined *Azi1^Gt/Gt^* mice using a peripheral blood micronuclei assay - a highly sensitive method for detecting *in vivo* DNA damage [53]. Whilst positive control *Mcph1^Gt/Gt^* mice show a marked increase in micronucleated erythrocytes as previously documented (http://www.sanger.ac.uk/mouseportal/phenotyping/MBGX/micronuclei/) [54], *Azi1* null mice show no such increase, suggesting there is no elevation in DNA damage in the peripheral blood of *Azi1* null mice (Figure S4L and M). Taken together, there is no gross evidence for chromosomal instability in *Azi1* null animals, neither *in vivo* nor in primary cell culture. Once again it is possible that the differences observed between siRNA knock-down and genetic null of *Azi1* are due to compensation for *Azi1* loss in the genetic null, as seen for the ciliogenic phenotypes observed.

### Azi1 function is not compensated in the sperm flagella resulting in male infertility

**Figure 6:**
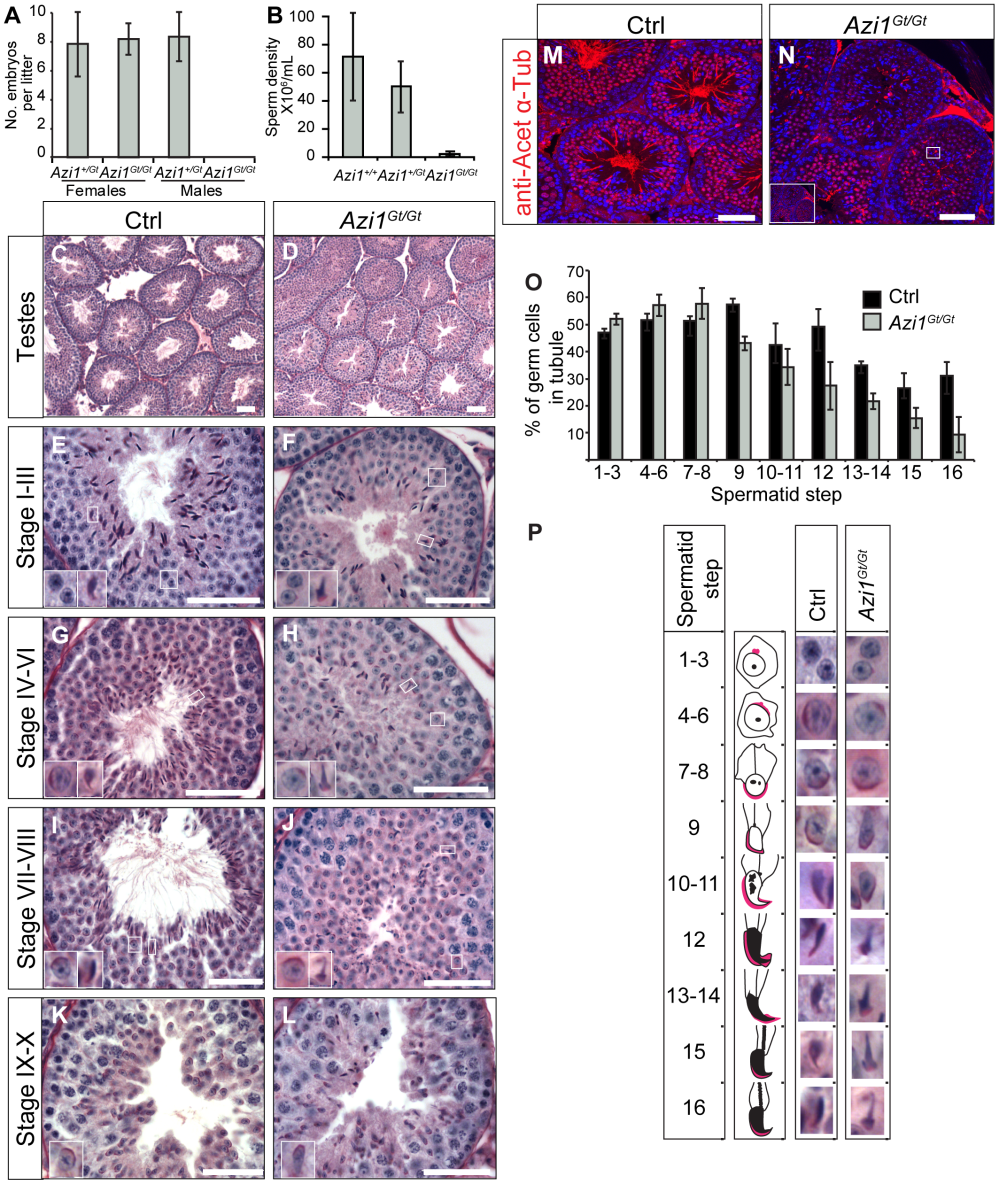
Loss of Azi1 cannot be compensated for in developing sperm flagella resulting in male infertility. (A) *Azi1^Gt/Gt^* males are infertile. Seven *Azi1^Gt/Gt^* males plugged *Azi1^+/Gt^* or *Azi1^+/+^* dams 25 times with no visible pregnancy or pups born. Shown is the mean litter size +/− standard deviation, n>3 animals, >5 litters per genotype. (B) Cauda sperm density in *Azi1^Gt/Gt^* and *Azi1^+/+^* mice, showing a 50-fold reduction in *Azi1^Gt/Gt^* males. Shown is mean +/− standard deviation. (C–L) PAS stained sections of control (Ctrl: *Azi1^+/+^* or *Azi1^+/Gt^*) and *Azi1^Gt/Gt^* adult testes. (C and D) *Azi1^Gt/Gt^* tubules show a reduced lumen diameter. (E–L) Stage-matched PAS stained tubules. Elongating spermatids from step 9, are found in reduced numbers in *Azi1^Gt/Gt^* testes (O). Flagella are clearly visible in control tubules (G, I and K) but are absent from stage-matched *Azi1^Gt/Gt^* tubules (H, J and L). *Azi1^Gt/Gt^* tubules appear disorganised, with mislocalisation and misorientation of elongated spermatids (F, H, J and L). Insets show enlargements of representative spermatids at each step of development (summarised in P). Many *Azi1^Gt/Gt^* spermatids show teratozoospermia from step 10 onwards. (M and N) *Azi1^+/+^* and *Azi1^Gt/Gt^* testes sections stained with anti-acetylated α-Tubulin, marking the flagella. Almost no flagella are seen in *Azi1^Gt/Gt^* tubules. Those remaining appear short and morphologically abnormal (Inset, N). (O) Spermatid numbers at each step of development as a percentage of total cells in the tubule. (P) Representative images of spermatids at each step of development taken from (C–K), with a schematic of spermatid morphology, highlighting the acrosome in pink. Spermatid numbers are reduced in *Azi1^Gt/Gt^* tubules from step 9, and morphological abnormalities in the spermatid head are seen from step 12, with a club-shaped morphology. Subtle morphological abnormalities are visible at step 10 with many *Azi1^Gt/Gt^* spermatids lacking the tapered end of the sperm head in. Scale bars represent 50 µm (C–L) or 100 µm (M and N).

We examined *Azi1* null mice carefully for adult-onset ciliopathic phenotypes to determine whether cilia formation and function was normal in all tissues. *Azi1* mutant males display complete infertility with no evidence of pregnancy or pups born from more than 25 plugged dams. In contrast, *Azi1^Gt/Gt^* female mice have normal litter numbers and sizes (Figure 6A). *Azi1^Gt/Gt^* males have reduced testes weight (*Azi1^Gt/Gt^*: 185 mg+/−23.9 mg vs. *Azi1^Gt/+^*: 127 mg+/−9.7 mg, *P*<0.05, Student's t-test) corresponding to a drastic reduction in sperm density (less than 2% of wild type) (Figure 6B). Male infertility is a common symptom of ciliopathies, often in conjunction with airway dysfunction and late-onset phenotypes including retinal degeneration, kidney cysts and obesity in mouse mutants of cilia genes [55], [56], [57]. Immunofluorescent and ultrastructural analysis of postnatal multiciliated airway epithelium revealed mutant cilia to be morphologically normal, consistent with the lack of chronic airway infections in these mice (Figure 3G–H and S3D–I). In aged cohorts of littermates, no signs of retinal degeneration were observed in *Azi1* null eyes either by ophthalmoscopic examination (data not shown) or histologically at six months (n = 7, Figure S5A–C). No cysts were observed in *Azi1* null kidneys aged 6 months or older (n = 6, Figure S5D–F), nor was any obesity observed in aged *Azi1^Gt/Gt^* mice (n = 8 at 3 months, n = 5 at 6 months, Figure S5G and H). In support of our cellular analysis, these *in vivo* studies of *Azi1* null mice demonstrate that *Azi1* is not required for cilia formation or function in general but is required for formation and function of the specialised cilia derivative, the sperm flagella.

### Spermiogenesis arrests at Step 9 in *Azi1* null tubules, with severe flagellar defects and teratozoospermia

To determine when spermatogenesis is disrupted in *Azi1* mutant mice, we examined periodic acid-Schiff (PAS) stained sections of testes. *Azi1^Gt/Gt^* mutant testes show a significant reduction in tubule lumen size (Lumen diameter: 15.1+/−1.2 µm in *Azi1^Gt/Gt^*, compared to 36.7+/−1.4 µm in *Azi1^+/+^*, mean +/− SEM, *P*<0.001, Student's t-test, n = 3), with a drastic reduction in the number of sperm flagella visible in the lumens of *Azi1^Gt/Gt^* testes (Figure 6C–L). Anti-acetylated α-Tubulin, which marks the flagellar axonemes, reveals that whilst control tubule lumens are filled with sperm flagella, almost none were detected in mutant tubules, and any flagella seen appeared shorter and morphologically abnormal, suggesting Azi1 is necessary for flagella formation (Figure 6M and N). Light microscopy revealed the pre-spermiogenic stages of spermatogenesis, up to Step 7–8 spermatids, appeared to be normal (Figure 6E–L, O–P). However, from Step 9, spermatid morphology is highly abnormal, with mutant elongating spermatids mislocalised and misorientated within the tubule. In addition to axonemal defects, mutant elongating spermatids also exhibit teratozoospermia, where sperm heads show an abnormal club-shaped nuclear morphology, as opposed to the normal hook-shaped head in wild type elongate spermatids (Figure 6E–L, P). Very few spermatids reach maturity and are successfully passed into the epididymis (Figure 6B, S6A and B). Importantly these phenotypes were observable from the first wave of spermatogenesis (Figure S6C–J). Whereas *Azi1^Gt/Gt^* tubules resemble *Azi1^+/+^* tubules at postnatal day 20 (P20) and P25 (Figure S6C–F), by P30, defects such as lack of flagella and misorientated spermatids displaying teratozoospermia become apparent. These results confirm observations in the adult mutant tubules that defects in spermiogenesis arise as the spermatids begin to elongate (P25–30) (Figure S6G–J).

To investigate whether the reduction in mature sperm was due to an increase in spermatid death in *Azi1^Gt/Gt^* tubules, we analysed levels of activated Caspase 3a and TUNEL staining, both indicators of apoptosis, in adult mutant tubules. *Azi1* mutant tubules showed increases in both activated Caspase 3a staining and TUNEL positive cells (Figure S6K–P). The restricted spatial distribution of activated Caspase 3a positive cells, combined with the cell counts showing a reduction in elongating spermatids (Figure 6O) indicate *Azi1^Gt/Gt^* spermatids undergo apoptosis from Step 9 onwards.

The germline is particularly sensitive to DNA damage and given the suggested role for *AZI1* in genome stability we considered whether *Azi1* null male infertility is due to an increase in DNA damage. Infertility due to defects in DNA damage response pathways generally presents as an early arrest in spermiogenesis, with spermatocytes not progressing through meiosis [58], [59], [60]. This is in contrast to the relatively late arrest in post-meiotic spermatogenesis seen in *Azi1* null mice, reminiscent of other ciliopathic mouse models [55], [56], [57], [61], [62]. To affirm that the arrest in spermatogenesis in *Azi1* mutant mice is not due to genome instability, we stained *Azi1* null testes with anti-γH2AX. In *Azi1* mutant tubules, we observed anti-γH2AX staining comparable to controls, emphasising that there is no increase in DNA double-stranded breaks in *Azi1* null testes (Figure S4N and O). Together with the previous *in vitro* and *in vivo* data (Figure S4), this shows there is no increase in DNA damage in the absence of *Azi1* under physiological conditions.

### Loss of *Azi1* results in multiple microtubule-dependent trafficking defects in mutant spermatids

**Figure 7:**
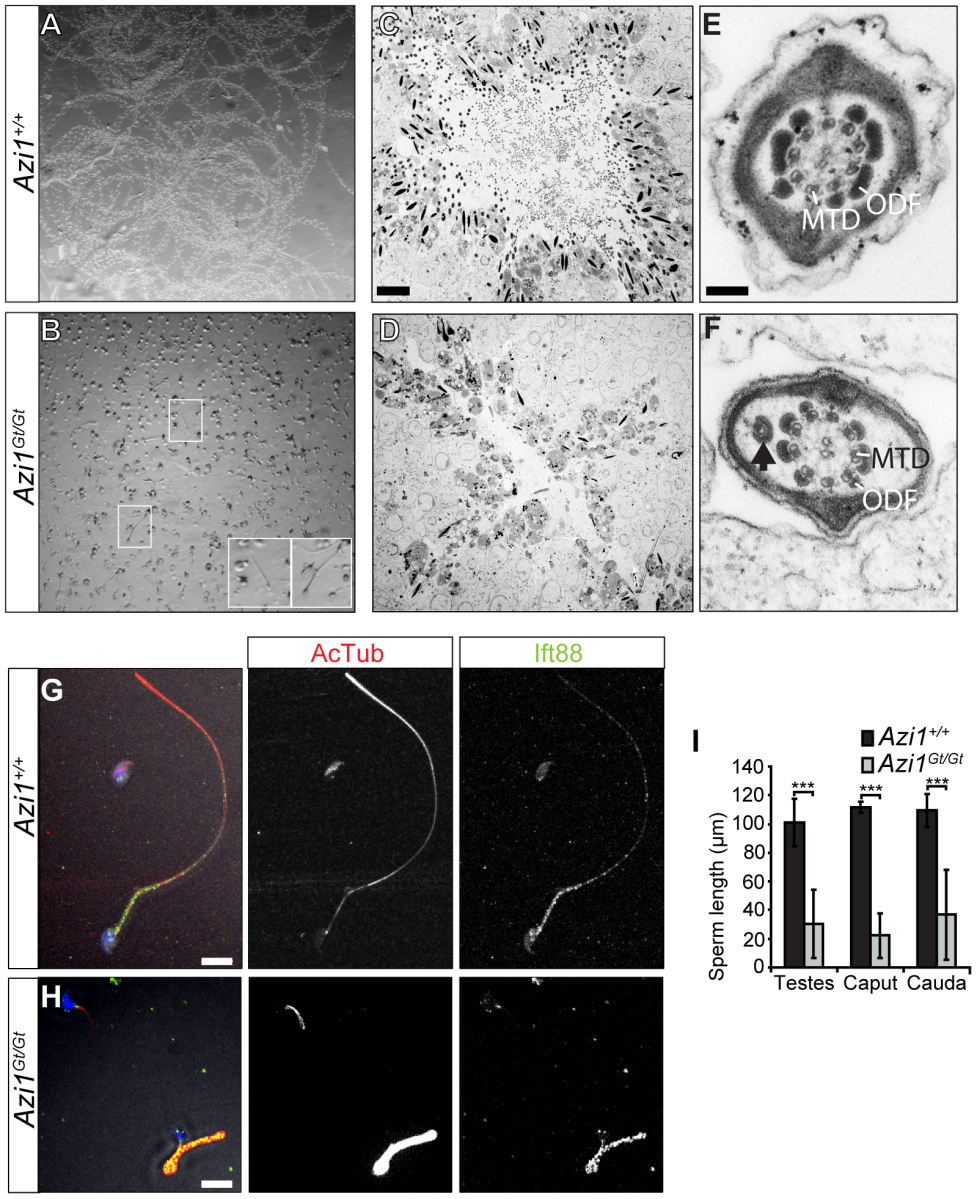
Remaining *Azi1^Gt/Gt^* sperm are immotile, with shortened flagella. (A and B) Sperm dissected from the cauda epididymis of *Azi1^+/+^* and *Azi1^Gt/Gt^* mice. *Azi1^+/+^* sperm was diluted 1∶5 in 1% methyl cellulose to slow movement to aid imaging. A projection of time points from high-speed video imaging shows the movement of *Azi1^+/+^* sperm was easily detectable (A). (B) No movement was detected in *Azi1^Gt/Gt^* sperm in methyl cellulose (not shown), so it was imaged undiluted without methyl cellulose. (B) A projection of time points shows the lack of movement of *Azi1^Gt/Gt^* sperm. Insets highlight rare sperm with intact flagella. (C and D) TEM of *Azi1^+/+^* and *Azi1^Gt/Gt^* tubules, showing an open lumen filled with sperm flagella in an *Azi1^+/+^* tubule (C), whereas *Azi1^Gt/Gt^* tubule lumens were filled with cellular debris and sperm flagella were difficult to find (D). (E and F) TEM of sperm flagella. (F) In *Azi1^Gt/Gt^* flagella that remain, the central axonemal structure appears generally normal, with the correct 9 + 2 microtubule structure in most remaining flagella. However evidence of abnormal trafficking was observed such as bulging membranes, ectopic outer dense fibres, or occasionally extra microtubules (arrow). MTD: microtubule doublet, ODF: Outer Dense Fibre (G–H). Sperm isolated from *Azi1^+/+^* and *Azi1^Gt/Gt^* testes was fixed and stained with anti-acetylated α-Tubulin and anti-Ift88. The number of *Azi1^Gt/Gt^* sperm was greatly reduced compared to *Azi1^+/+^*. All remaining *Azi1^Gt/Gt^* sperm had shortened and often kinked flagella, with increased anti-acetylated α-Tubulin in all flagella and abnormal distribution of anti-Ift88 staining in a subset of mutant flagella (H and I), suggestive of defects in IFT. (I) Quantification of flagella length from sperm isolated from the testes, the caput or the cauda epididymus, measured using both anti-acetylated α-Tubulin (anti-Acet α-tub) staining and transmitted light. Scale bars represent 10 µm (C, D, G and H) or 100 nm (E and F).

Cauda or caput epididymides from *Azi1* mutant mice only contained debris and degenerating sperm, none of which were motile (Figure 6B, 7A and B, Movie S2 and S3, and data not shown). Even in the testes, flagella were rarely observed by ultrastructure analysis of mutant tubules, which exhibited drastically reduced lumen diameters filled with vacuolar cells and proteinaceous cell debris, in contrast to the open, flagella filled wild type tubule lumens (Figure 7C and D). The rare flagella that remained showed evidence of abnormal trafficking, with swollen flagellar lumens and ectopic mistrafficked outer dense fibres, occasionally associated with microtubules (Figure 7E and F, S7). Remaining sperm were morphologically abnormal with flagella of severely reduced length (Figure 7G–I). The processes involved in extension of the mammalian sperm flagella are not well understood, but are thought to involve IFT-mediated trafficking [63], [64], [65]. Truncated *Azi1* mutant axonemes exhibit abnormal post-translational modifications of microtubules and irregular distributions of IFT trains, as marked by anti-Ift88 (Figure 7G and H). Extended axonemal structures are rarely detected by longitudinal TEM sections of mutant spermatids, instead occasional swollen shortened flagellar remnants with build-up of IFT cargo can be seen (Figure S7). Together these data suggest disruptions in regulated IFT account for the failure of flagellar axoneme extension.

**Figure 8:**
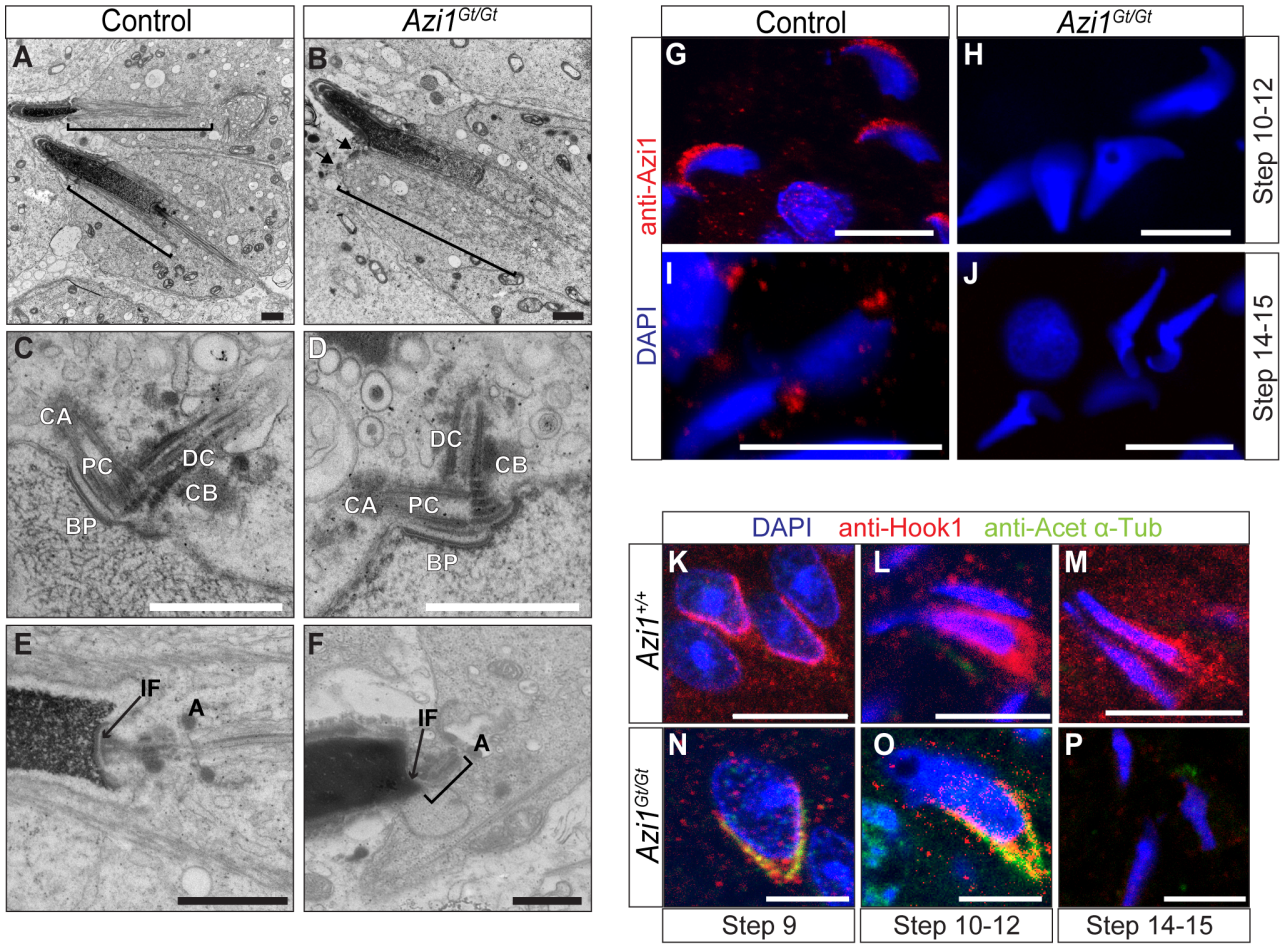
Mutant spermatids show defective manchette structure, and abnormal head morphologies, suggesting defects in IMT. (A and B) TEM of elongating spermatids, highlighting the manchette with brackets. The manchette forms in *Azi1^Gt/Gt^* spermatids, although it often appears kinked (Figure S7A), or is misnucleated away from the spermatid head (B, black arrows, also see Figure S7B). (C–F) Centrioles and the head tail coupling apparatus (HTCA) in early (C and D) or later stage (E and F) elongating spermatids from control (*Azi1^+/+^* or *Azi1^Gt/+^*) (C and E) or *Azi1^Gt/Gt^* testes (D and F). The centrioles implant normally at the early stages of *Azi1^Gt/Gt^* spermatid elongation, and some normal looking later stage HTCA can be found (Fig. S8D), although in many cases late stage HTCA are misaligned, with the implantation fossa or centrioles off-centre (F, brackets and arrow). (G–J) Elongating spermatids prepared by testes squashes from *Azi1^+/+^* or *Azi1^Gt/Gt^* animals were stained with anti-Azi1. Azi1 localises to the acrosome in Step 10–12 spermatids (G), then to punctae concentrated around centrosomes within the HTCA in control Step 13–14 spermatids (I). Importantly no Azi1 was detected in *Azi1^Gt/Gt^* spermatids, demonstrating the staining is specific. (K–P) Elongating spermatids in wax sections stained with anti-acetylated α-Tubulin and anti-Hook1. The manchette is not labelled by anti-acetylated α-Tubulin in wild type spermatids (K–M). In contrast, mutant manchettes display high levels of acetylated α-Tubulin (N and O), indicating altered microtubule dynamics. Hook1 is absent from late stage mutant spermatids, suggesting defects in intramanchette transport (IMT) (P). CA: centriolar adjunct, PC: proximal centriole, DC: Distal centriole, BP: basal plate, CB: centriolar body, IF: implantation fossa, A: annulus. Scale bars represent 2 µm (A and B), 1 µm (C–F), 10 µm (G–J) and 5 µm (K–P).

However, some of the abnormalities in *Azi1* mutant spermatid development, such as teratozoospermia cannot easily be explained by defects in IFT. These defects are first observed at step 9, when spermatids undergo a series of complex morphological changes, including nuclear remodelling and formation of the transient microtubule structure of the manchette [66], [67]. This microtubular “sleeve” structure surrounds the head and is assembled concurrently with the elongation and condensation of the spermatid nucleus, as well as growth of the centrosome-derived axoneme [68]. As in IFT, motor-driven trafficking along this track of microtubules delivers cargo from the Golgi-derived acrosome toward the centrosome and nascent sperm tail, in a process of intramanchette transport (IMT) [67]. Abnormal club shaped nuclei were previously observed in mutants with defects in manchette formation and function [69], [70]. Ultrastructural analysis revealed the manchette to be present in *Azi1* mutant spermatids but it often appears kinked, and is occasionally misnucleated away from the spermatid head (Figure 8A and B, S8A and B). Late stage spermatids exhibited abnormal nuclear morphologies, consistent with the histological analyses (Figure 6 and S6), often with detachment of the acrosome from the nucleus (Figure S8C–E). Formation of the sperm tail involves the migration and modification of a peripheral pair of centrioles to the caudal pole of the nucleus, opposite the acrosome, where they become lodged to form the neck or head-tail coupling apparatus (HTCA). In early spermatid differentiation, modifications to the proximal centriole, lodging of the centrioles into the implantation fossa of the nuclear membrane and formation of the centriolar adjunct appear grossly normal in *Azi1* mutant spermatids [71] (Figure 8C and D). A range of HTCA phenotypes are observed in later stage mutant spermatids, including misalignment of HTCA with the nucleus and/or displaced implantation fossa [72] (Figure 8E and F, S8F). Together, these results suggest *Azi1* may be required for maturation and functional integrity of the HTCA.

To understand these spermiogenic phenotypes observed in *Azi1* mutants, we examined Azi1 localisation during sperm development. Azi1 is found at the Golgi-derived acrosome (Step 10–12 spermatids: Figure 8G), then in the centrosome-containing HTCA at the flagellar base in later stage spermatids (Figure 8I). Importantly, no Azi1 staining is detected in any stage of mutant spermatids, confirming specificity of this Azi1 localisation (Figure 8H and J). This dynamic stage-specific redistribution of Azi1 is consistent with Azi1 undergoing IMT, although we failed to detect Azi1 specifically in the manchette, possibly due to dispersal of Azi1 below the detection threshold during IMT. This redistribution of Azi1 to the HTCA is consistent with a role in the maturation and functional integrity of this structure, and is reminiscent of enrichment of Azi1 around basal bodies upon ciliation in somatic cells.

We next examined localisation of an IMT cargo, Hook1, a coiled-coil protein implicated in vesicular transport which is mobilised progressively from the acrosome to HTCA during spermiogenesis [73], [74]. Post-translational modification of microtubules in the manchette are proposed to determine trafficking events by motors [74]. Manchette microtubules in wild type spermatids, although stabilised, are not labelled by the usual microtubule stabilising modifications, such as acetylation [75] (Figure 8K–M). However, strong anti-acetylated α-Tubulin staining is observed in Hook1-positive *Azi1* mutant manchettes at step 9–12 (Figure 8N and O). Subsequently, Hook1 is prematurely lost from Step 14–15 mutant manchettes (Figure 8M and P). These results suggest *Azi1* mutant spermatids exhibit altered microtubule dynamics and IMT cargo localisation. While intramanchette transport (IMT) is essential for both normal sperm head morphology and flagella formation [66], [73], [76], mouse mutations of components trafficked by IMT, like Hook1 [68], [69], [73] and RIM-BP3 [70], do not block extension of the flagellar axoneme completely.

These results suggest that loss of Azi1 disrupts the microtubule-based trafficking of both flagellar-directed IMT, and intraflagellar transport, resulting in both abnormal sperm head morphology together with the lack of flagella in *Azi1* mutant spermatids.

## Discussion

Genomic and proteomic studies on the centrosome/cilium complex (the “ciliome”) have estimated that thousands of gene products are involved in the formation and function of this important structure [8], [9]. Although many of the components are conserved across ciliates, it has generally been assumed that extrapolation from diverse genetic model organisms such as photosensitive, motile flagella of *Chlamydomonas* and ciliated mechanosensory chordotonal organs of *Drosophila* can be confidently extended to vertebrate cilia. In particular, there has been a growing trend of morpholino-based knock-down in the *D. rerio* model to functionally analyse putative ciliopathy candidate genes [13], [25]. Several recent mammalian cell culture-based knock-down screens have identified novel components involved in regulating cilia biology [10], [11], [12]. Facing a flood of next-generation sequencing detecting human disease variants, the daunting challenge is how best to investigate the requirement and function of novel and poorly characterised genes during development and disease.

**Figure 9:**
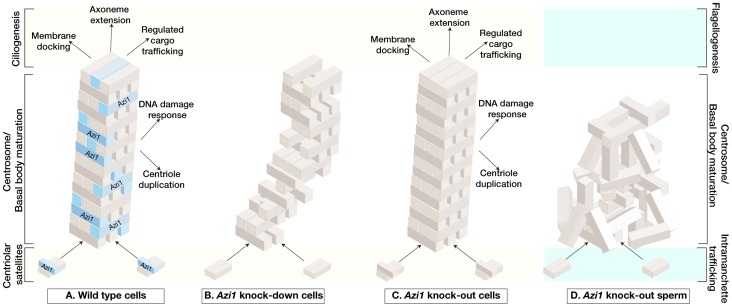
Model of acute versus chronic loss of mammalian *Azi1* results in distinct ciliary phenotypes. (A) In mammalian somatic cells, Azi1/Cep131 is a highly conserved and ubiquitously expressed coiled-coil protein found associated with centriolar satellites. During ciliogenesis it becomes enriched during basal body maturation and is found at the transition zone, or ciliary gate, suggesting it plays a role in membrane docking, axoneme extension and/or cargo delivery to nascent cilia. It is one component of a complex and dynamic network required to build and maintain cilia. (B) Transient Azi1/Cep131 knock-down (**acute depletion**) abruptly reduces levels of this protein and destabilises the entire ciliogenic network, resulting in lack of cilia in somatic cells. (C) Knock-out of Azi1/Cep131 (**chronic depletion**) in somatic cells allows the system to re-equilibrate the ciliogenic network through compensation and continue to build functional cilia. (D) Knock-out of Azi1/Cep131 (**chronic depletion**) in developing sperm cannot be compensated for and in its absence, the network never assembles properly. This results in defects in basal body maturation affecting axoneme extension and IFT-dependent cargo delivery necessary to build a functional flagellum. This *Azi1/Cep131* null sperm phenotype is similar to *Azi1* mutations in other model organisms.

Although *Azi1/Cep131* was predicted to be essential for cilia from studies in fly and zebrafish [24], [25], we present here detailed analysis of both acute loss (by siRNA) and chronic absence (by genetic mutation), and show that Azi1 has a conserved but non-essential role in mammalian ciliogenesis, and is essential for the formation of sperm flagella (Figure 9).

### Acute versus chronic lack of Azi1: Lessons for functional characterisation of putative human disease genes

We and others have shown transient knock-down of mammalian *Azi1* leads to a reduction in ciliogenesis [10]. Importantly, we show this *Azi1* knock-down ciliogenesis defect is rescued by overexpressing *Azi1-GFP*, emphasising the phenotype is “on-target” (Figure 1). Surprisingly, following genetic deletion of all gene function, cilia develop and function normally *in vivo* and in primary cells (Figure 3, 4, S3 and S5). Ruling out any sub-detectable “leakiness” of our *Azi1* gene-trap, transfection of *Azi1* siRNA into *Azi1^Gt/Gt^* MEFs does not affect ciliogenesis (Figure 5), suggesting that compensation for the lack of *Azi1* has occurred. It has been previously suggested that acute knock-down of proteins can give a more severe phenotype than long-term deletions due to compensation *in vivo* [50], [51]. We present the first demonstration of a lack of phenotype in null cells treated with siRNA against the gene of interest, proving this functional compensation exists.

In the absence of Azi1, mammalian cilia develop and function properly in all mouse tissues except for the developing sperm (Figure 3, 4 S3 and S5). *Azi1* is not essential for ciliogenesis and any involvement it may have in cilia biology can be compensated for in most tissues, aside from the modified cilia of the sperm flagella. *Azi1* null mice are born at sub-Mendelian ratios and a third of mutants appear to be lost before mid-gestation (Table 1), suggesting that a proportion of mutant embryos may fail to compensate for the loss of *Azi1* and die earlier in development. Several ciliopathy mouse models are also born at sub-Mendelian ratios, suggesting stochastic events may affect the requirement for ciliary proteins during embryogenesis [55], [56], [57], [77].

Our study highlights the importance of functional follow-up studies of siRNA data, and cautions against direct extrapolation of siRNA phenotypes to the genetic *in vivo* phenotype. On the other hand, acute knock-down by siRNA can expose roles for genes that would otherwise be overlooked due to compensation or redundancy in long-term deletion studies *in vivo*.

*Azi1* null male mice are infertile, suggesting the loss of Azi1 cannot be compensated for during spermiogenesis. *Azi1* null mice exhibit post-meiotic defects in spermatogenesis with misorientation and abnormal morphology of elongating and elongated spermatids, including teratozoospermia, from Step 9 of spermatid development onwards (Figure 6 and S6). Sperm flagella are mostly absent and any remaining axonemes are truncated and immotile, with swollen flagella lumens and evidence of mistrafficking of IFT components and cargo (Figure 7 and S7), suggesting Azi1 is required for IFT during the formation of the sperm flagella.

Additional roles for ciliary proteins in spermatid development outside the axoneme have been suggested, with parallels drawn between intraflagellar transport (IFT) and intramanchette transport (IMT) [67]. The abnormal club-shaped sperm head morphology is similar to mutants with defective manchettes [68], [69], [70]. *Azi1* mutant manchettes are structurally abnormal, and exhibit altered microtubule post-translational modifications, which are implicated in motor-selection for cargo delivery [74] (Figure 8 and S8). Altered expression of Hook1, an effector of IMT implicated in vesicular transport [74], is also observed in these mutant structures, suggesting the process of IMT is disrupted in *Azi1* mutants. Many motors and associated proteins, some linked to IFT, have been localised to the manchette and the testicular phenotype of *Ift88^orpk/orpk^* hypomorphic mice phenocopies the *Azi1*-dependent trafficking defects [67], [74].

Although the role of centriolar satellite or transition zone proteins in spermiogenesis is not well understood, it is tempting to draw parallels between the microtubule-based trafficking of centriolar satellites towards the centrosome/basal body of primary cilia, and the trafficking of proteins along the transient microtubular structure of the manchette to the highly specialised motile sperm flagellum. The dynamic relocalisation of Azi1 from the acrosome to the HTCA during spermatid development suggests Azi1 undergoes IMT, as this is the main transport route between these structures (Figure 8). Interestingly, components of the dynactin complex, which localise to centriolar satellites and is required for their peri-centrosomal localisation, have also been localised to the manchette [19], [47], [74], [78].

It is a conundrum for the cilia field as to why broadly expressed “core” cilia genes have clinically restricted phenotypes when mutated. This “tissue-specific” requirement is demonstrated by the limited phenotypes of late-onset ciliopathies, and many centriolar satellite and transition zone-specific mouse mutants display tissue specific ciliary phenotypes [56], [61], [62], [77], [79], [80]. In the case of the transition zone complex components, *Nphp4* and *Nphp1* null mutant mice display male infertility without kidney phenotypes, in contrast to the human disease in which patients have severe nephronophthisis [61], [62], [81]. This discrepancy could reflect differences in species and/or functional nature of the mutation, but the severity in humans could also reflect the mutational load in other components of the complex [82], [83]. Importantly primary cilia formation and function appears grossly normal in these mutants, like in our *Azi1* null mice, emphasising that compensation for function of key cilia genes is likely to be a recurrent theme given the central importance of primary cilia in mammalian development and patterning.

### Evolutionary function and divergence of the requirement for Azi1

Azi1/Cep131 is a conserved protein found in all ciliates except for nematodes, leading to the suggestion that Azi1 may be involved in cilia motility (Table S1) [46]. Alternatively, Azi1/Cep131 could be required to build a canonical nine-triplet centriole, as nematodes build specialized, non-canonical centrioles. However, an *Azi1/Cep131* orthologue is present in the *Toxoplasma gondii* genome which also build non-canonical centrioles [84]. Furthermore, knock-down of *Azi1/Cep131* in planaria does not affect centriole formation, but affects cilia motility [45]. While deletion mutations of *dila* in *D. melanogaster* [24] lead to defects in specialised “motile” mechanosenory Type I neurons and sperm, cilia do form with defects in mechanosensory neuronal cilia morphology characteristic of IFT mutants, suggesting defective trafficking. Similarly, *Azi1 D. rerio* morphants phenocopy IFT morphants with cilia forming but displaying tissue-specific reductions in length [25]. Interestingly, in trypanosomes TzAZI1 localises to the flagellar pocket, a dynamic endo-exocytic organelle implicated in membrane trafficking surrounding the flagellar base, and *TzAZI1* RNAi affects flagellar function, as opposed to its formation or maintenance [46]. These studies suggest a conserved trafficking function for *Azi1* in regulating ciliary-bound cargo.

We confirmed mammalian Azi1 localises to centriolar satellites and provide the first direct observation of Azi1 trafficking along microtubules, both towards and away from the centrosome (Figure 2G, Supplementary Movie 1), similar to the movement observed for PCM1-GFP [18]. The transport away from the centrosome may involve kinesin motors, and it has been shown that CEP290, which binds AZI1, interacts with both Dynactin components and the kinesin motor KIF3a [28], [79], supporting the theory that these proteins could undergo bidirectional trafficking along microtubules.

Centriolar satellites are thought to spatially restrict centrosomal access of proteins involved in basal body maturation and ciliogenesis [22]. While centriolar satellites and the key centriolar satellite protein, Pcm1, are not found in *Drosophila*, tight transcriptional control of *dila* mRNA limits expression to just before the onset of ciliogenesis where the protein can localise to the PCM [24]. Although the regulatory mechanisms are different, in both mammals and flies, it appears Azi1/dila recruitment is involved in centrosome to basal body maturation. In *D. melanogaster*, mutations in two other coiled-coil proteins YURI, conserved only among the *Drosophila* genus [85], [86], and UNC, also insect-specific but for which centriolar satellite protein OFD1 is proposed to be a functional orthologue [87] partially phenocopy *dila* mutants and genetically interact with *dila* [24]. These proteins are involved in the proper maturation and anchoring of the sperm basal bodies to the nuclear membrane [24], [86], [87]. Formation of *Drosophila* sperm flagella axoneme is unusual in that it is IFT-independent, forming instead in cytoplasmic cysts. Sperm axonemes do form in *dila* mutant flies, although the sperm display defective HTCA formation. This is reminiscent of the defects we observe in compromised integrity of HTCA in *Azi1* null sperm (Figure 8 and S8), although the requirement for IFT in mammalian sperm axonemal formation may explain the more severe IFT-based flagellar phenotypes observed in the mouse mutant spermatids.

Interestingly, similar to Ma *et al.* (2011) who showed transition zone localisation for DILA and UNC in *D. melanogaster* [24], we show Azi1 and PCM1 also localise to transition zone of primary cilium. While this manuscript was in preparation, OFD1 was also shown to localise to the transition zone [41]. Transition zone and centriolar satellite localisation has previously been described for CEP290, which was recently shown to interact with AZI1 [28], [38], [39]. Our data supports that redistribution of centriolar satellite proteins to the transition zone during ciliogenesis is of functional significance. It is possible that centriolar satellite-associated cargo is transported to the centrosome as it matures into a basal body with a functional transition zone ready to be trafficked into the cilium. Alternatively, Azi1/CEP290 could also have a role in the gating function at the transition zone, regulating protein content in the cilium, accounting for the IFT-like phenotypes reported in *dila Drosophila* mutants [24] and *Cep290 Chlamydomonas* mutants [42]. Given that centriolar satellites are scaffolds for controlling activity of ciliopathy-associated proteins [15], [22], it will be important to define the composition of these specific sub-complexes that move to the transition zone, and ask whether they are part of the compensation mechanism observed in *Azi1* null animals. If true, we propose that these interactions among the centriolar satellite proteins could extend to multi-allelic mutational load, including *AZI1*, in a subset of human ciliopathies with diverse clinical presentations beyond male infertility.

## Materials and Methods

### Cell culture and transfection

ShhLIGHT-II (ATCC, genetically modified NIH-3T3) and NIH-3T3 cells were maintained in DMEM (Life Technologies), hTERT-RPE cells were maintained in DMEM-F12 (Life Technologies), all supplemented with 1.5 g/L sodium bicarbonate (Sigma), 10% foetal calf serum, 5×10^8^ U/L penicillin and 11 mM streptomycin at 37°C, 5% CO_2_. Early passage MEFs were maintained in Optimem (Life Technologies) plus 0.5 mM beta-mercaptoethanol (Sigma), 10% foetal calf serum, 5×10^8^ U/L penicillin and 11 mM streptomycin at 37°C, 5% CO_2_, 3% O_2_. To induce ciliogenesis, serum was removed for 48 hours. ShhLIGHT-II cells were co-transfected with 25 nM Dharmacon OnTarget Plus siRNA and 1 µg/mL plasmid DNA with Dharmafect Duo (Dharmacon), serum was removed after 24 h and samples were analysed 72 hours after transfection. MEFs were co-transfected with 50 nM Dharmacon OnTarget Plus siRNA and 1.6 µg/mL plasmid DNA using the Invitrogen Neon, according to the manufacturer's protocol.

siRNA sequences used: Ctrl: 5′UGGUUUACAUGUCGACUAA3′; *Ift88* #3: 5′-CGGAGAAUGUUGAAUGUUU-3′; *Ift88* #4: 5′-GCUUGGAGCUUAUUACAUU-3′; *Azi1* 3′UTR Pool: Equimolar pool of: 5′-AGACACAGGGCUAAGGGUA-3′, 5′-CAGCUGUUCUAUAGUAAAA-3′, 5′-CCCUUGGGAUGACGAGCCA-3′ and 5′-GUGUCCAGGUCACGCUCCA-3′.

For live imaging of Azi1-GFP on Map4-RFP microtubules, NIH-3T3 cells were transduced with 30 particles per cell (PPC) of CellLIGHT MAP4-RFP BacMan 2.0 (Life Technologies), left for 24 hours then transfected with Azi1-GFP using Lipofectamine 2000 (Life Technologies) and imaged 24 hours later.

For DNA damage assays, MEFs were challenged with hydroxyurea (Sigma) at given concentrations for 18 hours. Alternatively, MEFs were irradiated in culture medium at 2Gy/minute using a Faxitron CellRad cabinet X-ray system (Faxitron Bioptics), cultured for 3 hours and then fixed and analysed.

### Generation of plasmids

*Azi1* was PCR amplified from mouse cDNA and TA cloned into pcDNA6.2-C-Em-GFP-GW-TOPO cDNA plasmid (Life Technologies). *Centrin2* was PCR amplified from mouse cDNA, adding EcoR1 and Sal1 sites. This was restriction enzyme cloned into pEGFP-N1 (Clontech).

### Western blot and densitometry

Cells or testes were homogenised in cell lysis buffer (Cell Signaling Technology) plus 1 mM phenylmethylsulfonyl fluoride (PMSF) (Thermo Scientific) and Complete Protease Inhibitor Cocktail (Roche), sonicated for 2×30 seconds. Testes extracts were concentrated with Amicon Ultra-0.5 mL 30 kDa centrifugal filters (Millipore). Samples were separated on Novex 3–8% Tris Acetate gels (Life Technologies) then transferred to Hybond nitrocellulose membrane (GE Healthcare), which were blocked in 5% milk. Membranes were incubated in primary antibodies (Table S4) washed, incubated with horse radish peroxidase (HRP) -conjugated secondaries (Table S5) and developed with Amersham ECL-plus western blotting detection system. Densitometry was performed using ImageJ.

### Immunocytochemistry

Cells were fixed in 4% paraformaldehyde/phosphate buffered solution (PFA/PBS) for 10 minutes at room temperature, or 4% PFA/PHEM (120 mM PIPES, 140 mM HEPES, 20 mM EGTA, 16 mM MgSO_4_, pH7) for 10 minutes at 37°C. Alternatively, pre-extraction was performed for 30 seconds on ice in 0.1 M PIPES pH 6.8, 2 mM EGTA and 1 mM MgSO_4_, then cells were fixed in ice cold methanol on ice for 10 minutes. Cells were blocked in 10% donkey serum/0.1% Triton-X in Tris buffered solution (TBS). Primary antibodies were added (Table S4), cells were washed then incubated in Alexa Fluor-conjugated secondaries (Table S5) and slides were mounted using Prolong Gold (Life Technologies).

### Generation of Azi1 mutant mice

*Azi1^+/Gt(CCOG35)Wtsi^* 129^Ola^ embryonic stem cells, which have a gene trap inserted into intron 2 of *Azi1*, were ordered from the Mutant Mouse Regional Resource Centre (MMRRC). ES cells were injected into C57BL/6J blastocysts and implanted into a recipient C57BL/6J female. These were backcrossed onto C57BL/6J for at least 5 generations for most analyses. Animals were maintained in SPF environment and studies carried out in accordance with the guidance issued by the Medical Research Council in “Responsibility in the Use of Animals in Medical Research” (July 1993) and licensed by the Home Office under the Animals (Scientific Procedures) Act 1986. Genotyping was performed using gene trap specific primers (GT Forward: 5′-GGTCCCGAAAACCAAAGAAG-3′ and GT Reverse: 5′-AGTATCGGCCTCAGGAAGATCG-3′) and primers to *Azi1* intron 2, spanning the insertion site which fail to amplify in the mutant (In Forward: 5′-GAGGAACCTGGGTGAGACCT-3′ and In Reverse: 5′-GCAGCAGATCTTTGGTCCAC-3′). Details of primers used for characterisation of the *Azi1^GT^* allele by RT-PCR and RT-qPCR are provided in Tables S2 and S3.

### Histology and immunohistochemistry

Tissue samples were collected, kidneys were fixed in 4% PFA/PBS, testes were fixed in Bouin's fixative, and eyes were fixed in Davidson's fixative according to standard protocols. Tissues were serially dehydrated and embedded in paraffin. Microtome sections of 8 µm thickness were examined histologically via haematoxylin and eosin (H&E) or periodic acid-Schiff (PAS) staining.

For immunofluorescent analysis, paraffin sections were dewaxed and re-hydrated via ethanol series. Antigen retrieval was performed by boiling the sections for 15 minutes in the microwave in citrate buffer. Sections were blocked in 10% donkey serum/0.1% Triton-X in PBS and primary antibodies were diluted in 1% donkey serum/PBS (Table S4). Slides were washed and incubated in Alexafluor conjugated secondary antibodies (Table S5), washed and mounted in ProLong Gold (Life technologies).

For immunohistochemistry, the same procedure was used, with the addition of one step after the re-hydration. Slides were immersed in 3% H_2_O_2_ in PBS for 20 minutes to block endogenous peroxidases. Slides were incubated in primary antibody, washed, then incubated in biotin-conjugated secondary antibody (Vector laboratories). This was detected using the Vector ABC kit and Vector NovaRed peroxidase substrate kit.

For TUNEL, after dewaxing, sections were incubated in 0.25% Triton-X and labelling was performed using Click-IT TUNEL staining kit following manufacturer's instructions (Life Technologies).

### LacZ staining using X-gal (5-bromo-4-chloro-3-indolyl-β-D-galactopyranoside)

E11.5 *Azi1^+/Gt^* embryos were fixed in 4% PFA/PBS for 20 minutes, rinsed in PBS and washed 3 times in detergent buffer (0.1 M phosphate buffer, 2 mM MgCl_2_, 0.1% sodium deoxycholate and 0.02% NP-40 (IGEPAL CA-630)). Embryos were then stained overnight in detergent buffer containing 50 mg/ml X-gal, 5 mM K_3_ and 5 mM K_4_ at 37°C, protected from light, washed twice in detergent buffer and post fixed overnight in 4% PFA.

### Sperm preparation

Testes, cauda and caput epididymides were dissected into M2 media (Invitrogen). For live imaging, sperm were imaged in M2 media or 1% methyl cellulose (Sigma), in capillary tubes (Vitrotubes Mountain Leaks) sealed with Cristaseal (Hawskley). Sperm counts were performed on sperm from the cauda epididymides, diluted in H_2_O using a haemocytometer, only counting intact sperm (with both head and tail). For fixed samples, either sperm spreads or testes squashes were prepared. For sperm spreads, testes were placed through a 100 µm nylon mesh (BD Biosciences). Sperm from the caput epididymus and testes were then placed on a 20–40% Percoll gradient (GE Healthcare) and spun at 3,000 g for 10 minutes. The sperm were spread on Poly-D-lysine slides (BD Biosciences) and fixed 4% PFA/PBS. Cauda sperm was spread directly on Poly-D-Lysine slides and fixed with 4% PFA/PBS. In all cases, sperm were permeabilised with 0.4% Triton-X in PBS and immunofluorescence was performed as described. For testes squashes, tubules were dissected as described [88], placed on a slide and squashed with a coverslip. Slides were snap frozen in liquid nitrogen, coverslips removed and samples fixed and permeabilised by 10 minutes in −20°C methanol, 30 seconds in acetone and then 15 minutes in 4% PFA/PBS. Immunofluorescence was then performed as described.

### Nasal brushing

Nasal brushing was performed as described [89]. Cells were fixed for 30 minutes on ice in 4% PFA, cytospun onto slides, fixed with −20°C methanol for 10 minutes then immunofluorescence was performed as described.

### Transmission electron microscopy

Samples were dissected into PBS. Samples were fixed in 2% PFA/2.5% gluteraldehyde/0.1 M Sodium Cacodylate Buffer pH7.4 + 0.04% CaCl_2_. Testes capsules were removed prior to immersion in fix. After 30 minutes at room temperatures, samples were cut into 1 mm cubes and fixed overnight or longer at 4°C. Tissue was rinsed in 0.1 M sodium cacodylate buffer, post-fixed in 1% OsO_4_ (Agar Scientific) for one hour and dehydrated in sequential steps of acetone prior to impregnation in increasing concentrations of resin (TAAB Lab Equipment) in acetone followed by 100%, placed in moulds and polymerised at 60°C for 24 hours.

Ultrathin sections of 70 nm were subsequently cut using a diamond knife on a Leica EM UC7 ultramicrotome. Sections were stretched with chloroform to eliminate compression and mounted on Pioloform filmed copper grids prior to staining with 1% aqueous uranyl acetate and lead citrate (Leica). They were viewed on a Philips CM100 Compustage Transmission Electron Microscope with images collected using an AMT CCD camera (Deben).

### Micronuclei assay

Mice were tail tipped and blood was collected using a microhematocrit capillary tube with heparin coating (Globe Scientific) into heparin (Sigma). This was fixed in super-chilled methanol. Saline was added and then blood was pelleted at 600 g for 5 minutes. Pellets were treated with RNAseA and anti-CD71primary antibody (Lifespan Biosciences). Cells were incubated on ice for 30 minutes then at room temperature for 30 minutes. Propidium iodide was added and flow cytometry was performed on a FACScalibur (BD Biosciences). Data was analysed using FlowJo software (v7.6.1, Tree Star) as described by [53].

### Imaging and image analysis

The initial siRNA screen imaging was carried out on the Olympus Scan^R^ microscope, imaging 16 frames per well. Image analysis, including identification and counting of cells and cilia was performed using the Olympus Scan^R^ Analysis Software.

Confocal images were captured with a Nikon A1R confocal microscope, comprising a Nikon Eclipse TiE inverted microscope and four laser modules: 405 (laser diode), 457, 488, 514 (multiline Argon) 561 (diode-pumped solid-state) and 638 nm (laser diode). For live imaging of Azi1-GFP and MAP4-RFP, a Zeiss Axiovert 200 fluorescence microscope was used equipped with 100×/1.4 plan apochromat objective (Carl Zeiss, Welwyn, UK), Lambda LS 300W Xenon source with liquid light guide and 10-position excitation, neutral density and emission filterwheels (Sutter Instrument, Novato, CA), ASI PZ2000 3-axis XYZ stage with integrated piezo Z-drive (Applied Scientific Instrumentation, Eugene, OR) and a Photometrics Coolsnap HQ_2_ CCD camera (Roper Scientific, Tucson, AZ). Brightfield images were captured with a Coolsnap HQ CCD camera (Photometrics Ltd, Tucson, AZ) on a Zeiss Axioplan II fluorescence microscope with Plan-neofluar objectives (Carl Zeiss, Welwyn Garden City, UK). Colour additive filters (Andover Corporation, Salem, NH) installed in a motorised emission filter wheel (Prior Scientific Instruments, Cambridge, UK) were used sequentially to collect red, green and blue images that were then superimposed to form a colour image.

For live imaging of sperm, a Qimaging Retiga camera running at 30 frames per second (bin 2×2 half frame) captured image sequences with a 5× objective at zoom 5 on a Nikon AZ100 macroscope. Still figures show all time points superimposed into one image to depict the movement, or lack thereof, during the movie.

Apart from the initial screen, image analysis including intensity profiling was performed in ImageJ.

### Statistics

Throughout *P*<0.05 is considered significant. Statistics were carried out in Microsoft Excel or GraphPad Prism (La Jolla, CA) and the test used is specified in the text/figure legend.

## Supporting Information

Figure S1 Mouse and human AZI1 localise to centriolar satellites and the transition zone, but are not required for centriolar satellite formation. Mouse and human AZI1 localises to centriolar satellites and the transition zone in NIH-3T3 cells. (A) Co-staining with anti-Azi1 SF91 and anti-Pcm1 shows mouse Azi1 localises to centriolar satellites in NIH-3T3 cells. Enlargements to the right highlight the co-localisation of Pcm1 (middle) and Azi1 (bottom). (B) Localisation of AZI1 to the transition zone in hTERT-RPE-1 cells (Figure 2A–D) was confirmed with a second anti-AZI1 antibody (SF91). (C and D) Co-staining of anti-Azi1 (SF91) with anti-polyglutamylated Tubulin (PGT) (C), or anti-Nphp1 and anti-acetylated α-Tubulin (AT) (D) shows mouse Azi1 localises to the transition zone. Enlargement of cilia to the right show individual channels, with an intensity profile below confirming that Azi1 localisation to the transition zone. (E–H) Mouse ShhLIGHT-II fibroblast cells were co-transfected with siRNA (a non-targeting control siRNA (Ctrl), siRNA targeting *Ift88*, (Ift88 #3) or a pool of four siRNAs targeting the 3′ UTR of *Azi1* (*Azi1* 3′UTR), along with plasmids encoding either *eGFP* or *Azi1-GFP* (which lacks the 3′UTR of *Azi1*). Cells were stained with anti-Pcm1 (red) and GFP Booster (green: Chromotek) (E–G). (H) Pcm1 localisation in transfected cells was classified as “strong pericentrosomal” (filled arrowhead), “weak pericentrosomal” (open arrow) or “dispersed” (open arrowhead/triangle). There was no difference in Pcm1 localisation upon *Azi1* siRNA addition. Magnified panel below G highlights the co-localisation of Azi1-GFP with Pcm1. Scale bars represent 10 µm (A–D) or 20 µm (E–G).
(TIF)

Figure S2 Azi1 domain structure and further characterisation of *Azi1* transcript levels in *Azi1^Gt/Gt^* mice. (A) Schematic showing *Azi1* gene structure and gene trap insertion. Exons are shown as boxes with translated transcript shaded. Arrows indicate primers used to characterise the mRNA expression in *Azi1^Gt/Gt^* mice. SA: Splice acceptor, T2A: self-cleaving peptide, pA: polyA. (B) Schematic of the predicted domain structure of Azi1 (ENSMUSP00000101834.1, predicted domains from ENSEMBL mouse release 72). Red arrow indicates the predicted site of truncation in *Azi1^Gt/Gt^* mice. The truncated product lacks all the predicted domains, including highly conserved coiled-coil domains. The epitope of the Azi1 SF91 and Abcam ab110018 antibodies are indicated. (C) RT-PCR of exons 10–24 of *Azi1* in kidney (K) and ovary (O) of *Azi1^+/+^* and *Azi1^Gt/Gt^* mice. No expression of exons 10–13 was detected. Some reduced-level expression of exons 20–24 was detected, consistent with non-coding transcripts predicted by ENSEMBL (mouse release 72). (D and E) qPCR on testes cDNA of exons 7–8 of *Azi1* (D), showing negligible expression in *Azi1^Gt/Gt^* mice (0.3% of wild type), and of exons 24–26 of *Azi1* (E), again showing low levels of expression in *Azi1^Gt/Gt^* mice (1.6% of wild type).
(TIF)

Figure S3 Azi1 is not required for centriolar satellite or transition zone formation, nor basal body docking. (A–C) Pcm1 localisation (green) was analysed in *Azi1^+/+^* and *Azi1^Gt/Gt^* MEFs, with the centrosome marked with anti-γ Tubulin (red, anti-γ tub) (A and B). Pcm1 localisation was classified as “strong pericentrosomal” (filled arrow), “weak pericentrosomal” (open arrow) or “dispersed” (open headed arrow/triangle) (C). No difference in Pcm1 localisation was observed between *Azi1^+/+^* and *Azi1^Gt/Gt^* MEFs. Shown is mean +/− standard deviation (n = 3). (D–I) TEM of *Azi1^+/+^* and *Azi1^Gt/Gt^* motile multiciliated epithelial cells lining the adult trachea. (D and G) Basal bodies properly dock in the *Azi1^Gt/Gt^* trachea and appendages are formed normally (arrowheads). (E and H) Transition zones form and appear morphologically normal in the *Azi1^Gt/Gt^* trachea (brackets). (F and I) Axonemes of motile tracheal cilia appear normal with the expected (9+2) microtubule morphology, and the presence of inner and outer dynein arms (arrows and arrowheads, respectively). Scale bars represent 50 µm (A and B), 2 µm (D, E, G and H) or 100 nm (F and I).
(TIF)

Figure S4 *Azi1^Gt/Gt^* mutant mice show no gross increases in DNA damage. DNA damage, measured by anti-γH2AX staining, was assessed in *Azi1^+/+^* and *Azi1^Gt/Gt^* MEFs without challenge (A and F), challenged with hydroxyurea (HU) (B–D and G–I) or challenged with 3 Gray (Gy) of ionising radiation (I,J). (K) The intensity of γH2AX staining is plotted in arbitrary units (AU). There is no significant difference in γH2AX staining between *Azi1^+/+^* and *Azi1^Gt/Gt^* MEFs when unchallenged, or challenged with up to 2 mM HU or 3 Gy ionising radiation. *Azi1^Gt/Gt^* MEFs are more sensitive to high concentrations (5 mM) HU. * *P*<0.05, ANOVA, n = 3. Shown is mean + STD. (L–M) The percentage of micronuclei in peripheral blood was assessed by flow cytometry. (M) Example FACS data gated to show erythrocytes (Ery, CD71 negative) and reticulocytes (ret, CD71 positive), the high PI containing cells are micronucleated (MN). (N) Plot of the percentage of micronucleated erythrocytes. *Mcph1^−/−^* mice show increased numbers of micronucleated erythrocytes, as previously documented (http://www.sanger.ac.uk/mouseportal/phenotyping/MBGX/micronuclei/) (M). *Azi1^Gt/Gt^* mice show no change in numbers of micronucleated erythrocytes, suggesting no increased DNA damage *in vivo*. (N and O) DNA damage was assessed in the testes by anti-γH2AX staining. As DNA double-stranded breaks occur during meiosis, γH2AX staining occurs normally in certain cell types such as in foci in some spermatogonia and preleptotene to zygotene spermatocytes, in the XY body of pachytene spermatocytes and in the nucleus of round spermatids [90]. Comparing stage matched tubules (assessed by serial PAS sections), the *Azi1^Gt/Gt^* testes showed no increase in γH2AX staining. In both *Azi1^+/+^* and *Azi1^Gt/Gt^* tubules, anti-γH2AX stains the XY body (white arrows) of pachytene spermatocytes (PS) and, less intensely, round spermatids (RS). It is absent from elongating spermatids (ES). Scale bars represent 50 µm (A–J) or 25 µm (N and O).
(TIF)

Figure S5 No late-onset ciliopathy phenotypes are observed in *Azi1^Gt/Gt^* mice. (A–C) Eyes of seven mutant mice at 6 months of age were directly examined by opthalmoscope and no retinal degeneration was observed (data not shown). This was confirmed histologically on H&E stained wax sections in four mutants. Shown are representative sections of the retina from wild type (A) or two *Azi1* null mice (B and C). (D–F) Kidneys were taken from seven mutants aged 6 months or more, and no kidney cysts were seen. This was confirmed by H&E stained sections for four mutants. (E and F) Representative kidney sections from 6 months old *Azi1^Gt/Gt^* mice, and *Azi1^Gt/+^* littermate, showing no cysts in the mutant kidneys. (G and H) The weight of male (G) and female (H) *Azi1^Gt/Gt^* mice is no different to *Azi1^+/+^* and *Azi1^Gt/+^* mice at 1–9 months old. Scale bars represent 50 µm.
(TIF)

Figure S6 *Azi1^Gt/Gt^* sperm morphological defects begin during spermatid elongation, leading to increased apoptosis in adult testes. (A–B) H&E stained sections of adult epididymides, showing a dramatic reduction in the number of sperm present in the *Azi1^Gt/Gt^* epididymis. (C–J) PAS stained sections of control (C, E, G and I) or *Azi1^Gt/Gt^* (D, F, H and J) tubules from P20 (C and D), P25 (E and F), P30 (G and H) and P35 (I and J) mice, showing the first coordinated wave of spermatogenesis. *Azi1^Gt/Gt^* tubules resembled wild type at P20 and P25, suggesting spermatogenesis progresses normally until spermatid elongation. By P30, the abnormalities seen in adult testes (Figure 6C–L) become apparent in the *Azi1^Gt/Gt^* tubules, such as a lack of sperm tails (compare lumens in H and J to G and I), disorganisation of the tubules, as demonstrated by the misorientation and mislocalisation of elongating spermatids in H and J, and morphologically abnormal spermatid heads (insets). (K and L) Sections of adult testes anti-activated Caspase 3a (anti-Act Casp3), showing a significant increase in the number of cells undergoing apoptosis in *Azi1^Gt/Gt^* tubules (marked by white arrowheads). Quantified in (O) (*P*<0.0001, n = 3, Mann Whitney U test). (M and N) Adult testes sections labelled with TUNEL, showing an increase in the number of dying cells in *Azi1^Gt/Gt^* tubules (marked by white arrowheads), although this is not quite statistically significant. Quantified in (P) (*P* = 0.051, n = 3, Mann Whitney U test). Scale bars represent 50 µm (A–J) or 100 µm (K–N).
(TIF)

Figure S7 *Azi1* null spermatids exhibit IFT-like trafficking defects. (A) In control testes, an elongated spermatid with progressively condensed nucleus with well-defined basal plate where the centriolar complex with extending flagella (arrowhead) has lodged, forming the head-tail connecting apparatus (HTCA). (B, B′) Serial sections of *Azi1^Gt/Gt^* elongated testicular spermatid with abnormally swollen and truncated flagellar structure (arrowhead) with no clear axoneme beyond the electron-dense annulus. (B,C,C′) Accumulation of IFT-cargo microtubules visible at distal tip of structure (red arrow), with associated outer dense fibres. C′ is higher magnification view of C, another distal cytoplasmic accumulation of microtubules and outer dense fibres. (D) Absence of discernible flagellar structures associated with HTCAs and annuli is the primary phenotype of *Azi1* mutant spermatids. Scale bar represents 500 nm (A, B, B′, C and D) or 100 nm (C′).
(TIF)

Figure S8 *Azi1* mutant spermatids show defects in manchette structure, nuclear abnormalities and HTCA alignment. (A and B) Further examples of *Azi1* mutant manchette defects, including kinking (A arrows and brackets) and misnucleation (open arrow) (B). (C–E) Defects in intramanchette transport can lead to abnormalities in nuclear morphology as well as acrosome defects (arrowheads C and E). (F) A further example of a misaligned HTCA, as in Figure 8H, with the implantation fossa off-centre (short arrow). Brackets mark ectopic microtubules, possibly ectopic manchette.
(TIF)

Movie S1 Live imaging of Azi1-GFP in an NIH 3T3 cell. Arrows mark Azi1-positive centriolar satellites moving towards (white) or away from (yellow) from the centrosome (where the bulk of Azi1-GFP lies).
(AVI)

Movie S2 Fast imaging of wild sperm motility.
(AVI)

Movie S3 Fast imaging of *Azi1* null sperm, showing complete immotility, although twitching proves the sperm is alive.
(AVI)

Table S1 Conservation of *AZI1/CEP131*.
(XLSX)

Table S2 Primers for RT-PCR of *Azi1*.
(XLSX)

Table S3 Primers for qRT-PCR.
(XLSX)

Table S4 Primary antibodies.
(XLSX)

Table S5 Secondary antibodies.
(XLSX)
