# Supplementary figures and images for: Acute Versus Chronic Loss of Mammalian Azi1/Cep131 Results in Distinct Ciliary Phenotypes
Source: PLoS Genet. 2013 Dec 26;9(12):e1003928. doi: 10.1371/journal.pgen.1003928 (PMC3887133; doi:10.1371/journal.pgen.1003928)

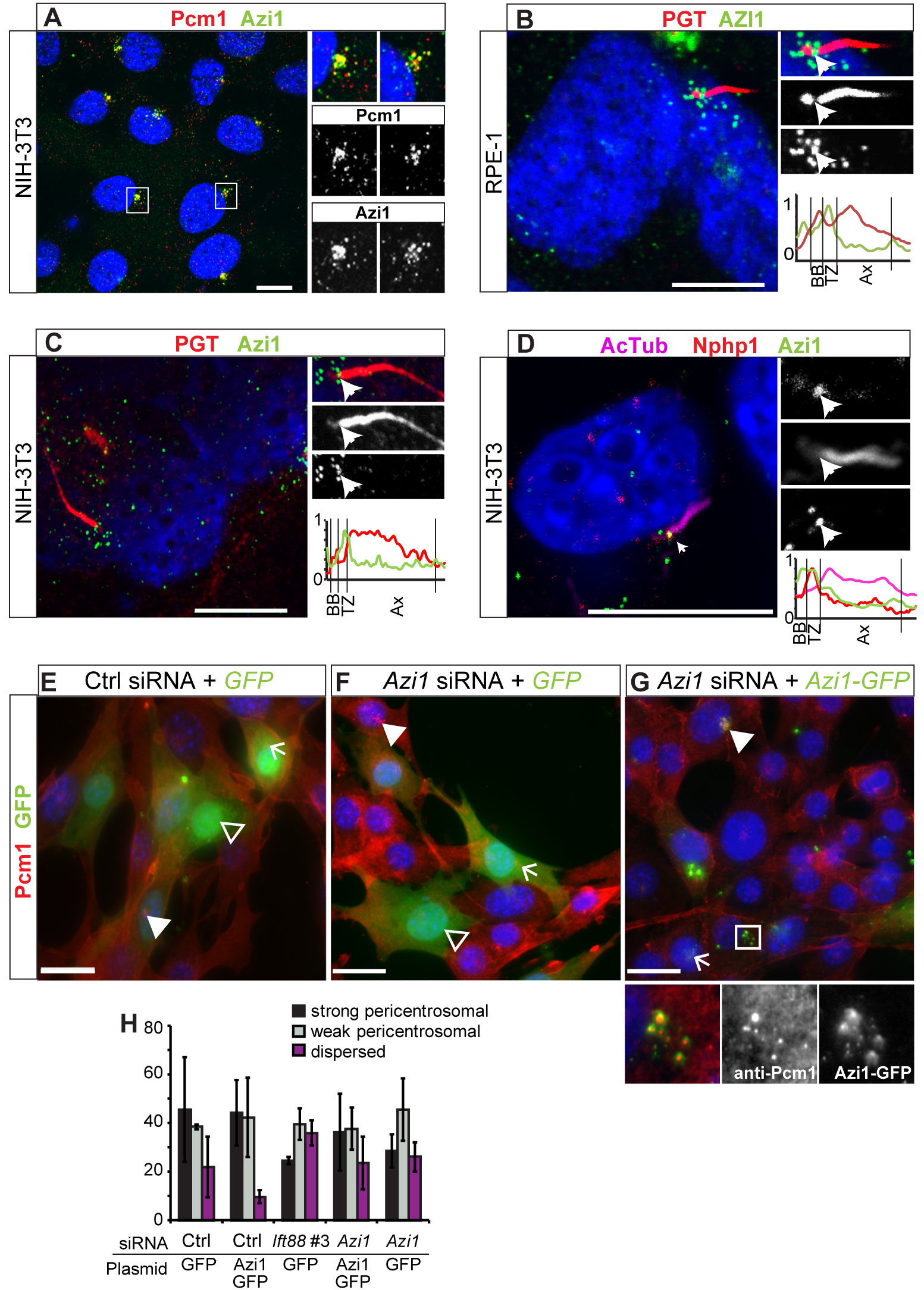

Supplement: Figure S1 — Mouse and human AZI1 localise to centriolar satellites and the transition zone, but are not required for centriolar satellite formation. Mouse and human AZI1 localises to centriolar satellites and the transition zone in NIH-3T3 cells. (A) Co-staining with anti-Azi1 SF91 and anti-Pcm1 shows mouse Azi1 localises to centriolar satellites in NIH-3T3 cells. Enlargements to the right highlight the co-localisation of Pcm1 (middle) and Azi1 (bottom). (B) Localisation of AZI1 to the transition zone in hTERT-RPE-1 cells (Figure 2A–D) was confirmed with a second anti-AZI1 antibody (SF91). (C and D) Co-staining of anti-Azi1 (SF91) with anti-polyglutamylated Tubulin (PGT) (C), or anti-Nphp1 and anti-acetylated α-Tubulin (AT) (D) shows mouse Azi1 localises to the transition zone. Enlargement of cilia to the right show individual channels, with an intensity profile below confirming that Azi1 localisation to the transition zone. (E–H) Mouse ShhLIGHT-II fibroblast cells were co-transfected with siRNA (a non-targeting control siRNA (Ctrl), siRNA targeting Ift88, (Ift88 #3) or a pool of four siRNAs targeting the 3′ UTR of Azi1 (Azi1 3′UTR), along with plasmids encoding either eGFP or Azi1-GFP (which lacks the 3′UTR of Azi1). Cells were stained with anti-Pcm1 (red) and GFP Booster (green: Chromotek) (E–G). (H) Pcm1 localisation in transfected cells was classified as “strong pericentrosomal” (filled arrowhead), “weak pericentrosomal” (open arrow) or “dispersed” (open arrowhead/triangle). There was no difference in Pcm1 localisation upon Azi1 siRNA addition. Magnified panel below G highlights the co-localisation of Azi1-GFP with Pcm1. Scale bars represent 10 µm (A–D) or 20 µm (E–G). (TIF) [file pgen.1003928.s001.tif]

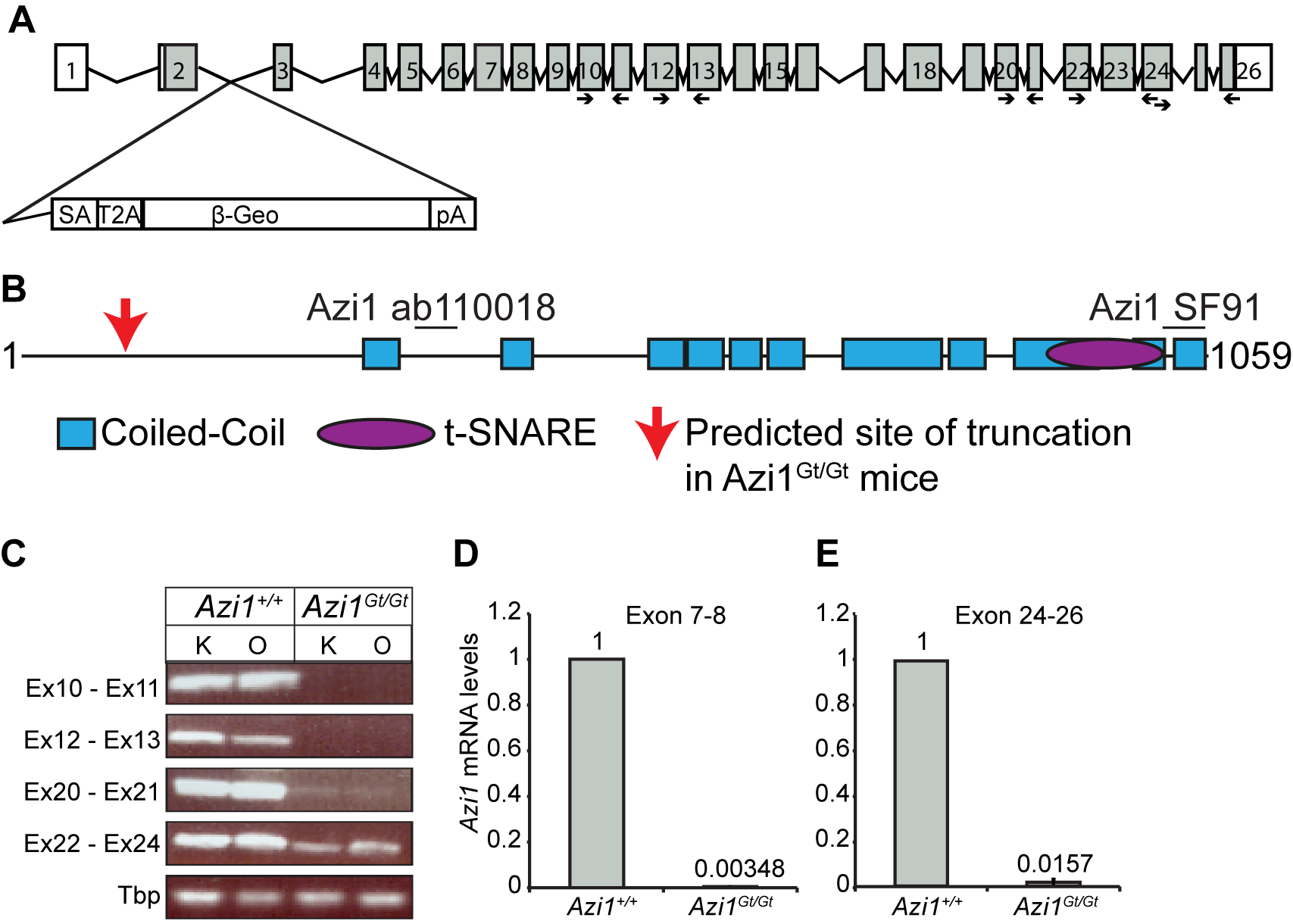

Supplement: Figure S2 — Azi1 domain structure and further characterisation of Azi1 transcript levels in Azi1Gt/Gt mice. (A) Schematic showing Azi1 gene structure and gene trap insertion. Exons are shown as boxes with translated transcript shaded. Arrows indicate primers used to characterise the mRNA expression in Azi1Gt/Gt mice. SA: Splice acceptor, T2A: self-cleaving peptide, pA: polyA. (B) Schematic of the predicted domain structure of Azi1 (ENSMUSP00000101834.1, predicted domains from ENSEMBL mouse release 72). Red arrow indicates the predicted site of truncation in Azi1Gt/Gt mice. The truncated product lacks all the predicted domains, including highly conserved coiled-coil domains. The epitope of the Azi1 SF91 and Abcam ab110018 antibodies are indicated. (C) RT-PCR of exons 10–24 of Azi1 in kidney (K) and ovary (O) of Azi1+/+ and Azi1Gt/Gt mice. No expression of exons 10–13 was detected. Some reduced-level expression of exons 20–24 was detected, consistent with non-coding transcripts predicted by ENSEMBL (mouse release 72). (D and E) qPCR on testes cDNA of exons 7–8 of Azi1 (D), showing negligible expression in Azi1Gt/Gt mice (0.3% of wild type), and of exons 24–26 of Azi1 (E), again showing low levels of expression in Azi1Gt/Gt mice (1.6% of wild type). (TIF) [file pgen.1003928.s002.tif]

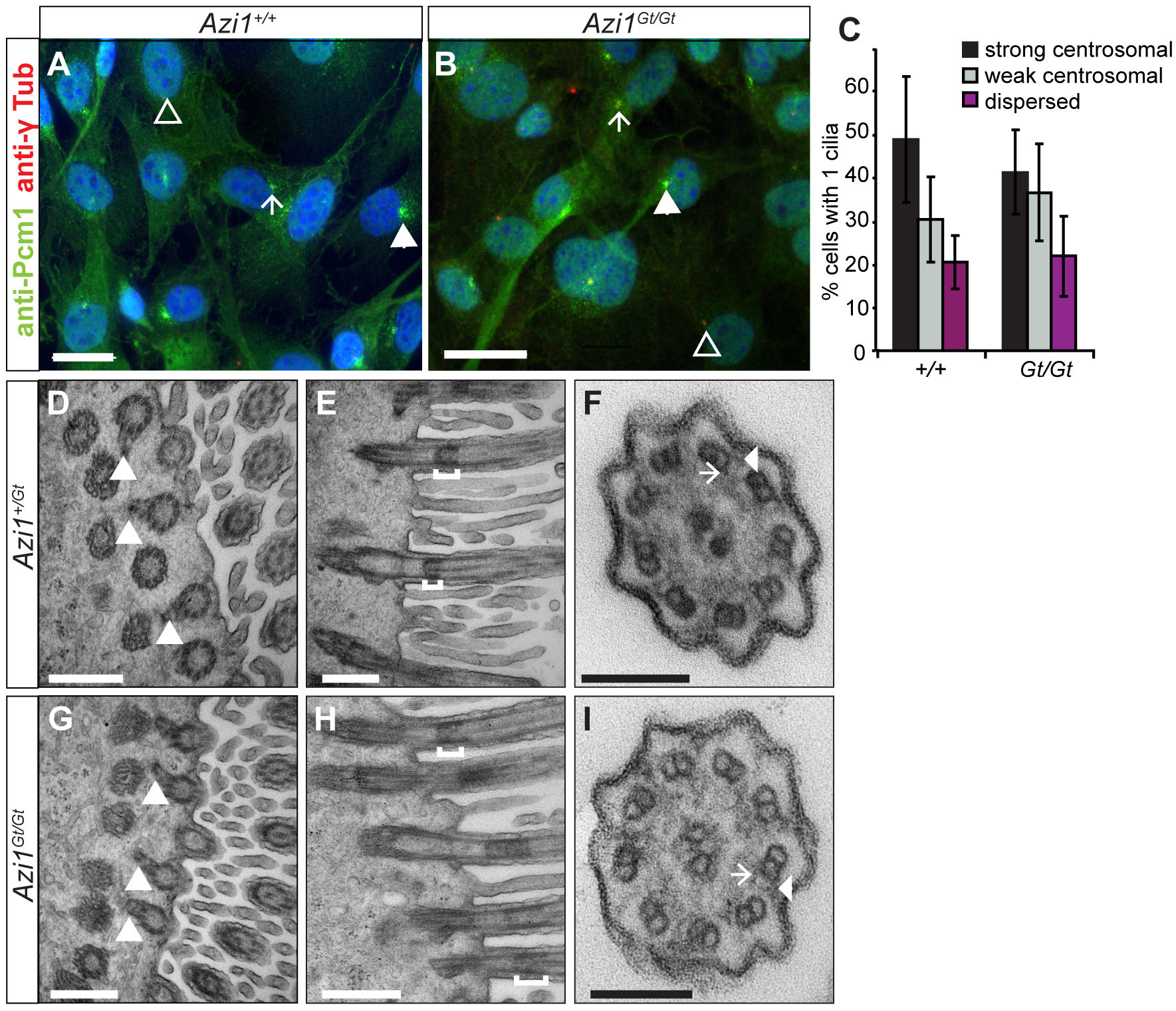

Supplement: Figure S3 — Azi1 is not required for centriolar satellite or transition zone formation, nor basal body docking. (A–C) Pcm1 localisation (green) was analysed in Azi1+/+ and Azi1Gt/Gt MEFs, with the centrosome marked with anti-γ Tubulin (red, anti-γ tub) (A and B). Pcm1 localisation was classified as “strong pericentrosomal” (filled arrow), “weak pericentrosomal” (open arrow) or “dispersed” (open headed arrow/triangle) (C). No difference in Pcm1 localisation was observed between Azi1+/+ and Azi1Gt/Gt MEFs. Shown is mean +/− standard deviation (n = 3). (D–I) TEM of Azi1+/+ and Azi1Gt/Gt motile multiciliated epithelial cells lining the adult trachea. (D and G) Basal bodies properly dock in the Azi1Gt/Gt trachea and appendages are formed normally (arrowheads). (E and H) Transition zones form and appear morphologically normal in the Azi1Gt/Gt trachea (brackets). (F and I) Axonemes of motile tracheal cilia appear normal with the expected (9+2) microtubule morphology, and the presence of inner and outer dynein arms (arrows and arrowheads, respectively). Scale bars represent 50 µm (A and B), 2 µm (D, E, G and H) or 100 nm (F and I). (TIF) [file pgen.1003928.s003.tif]

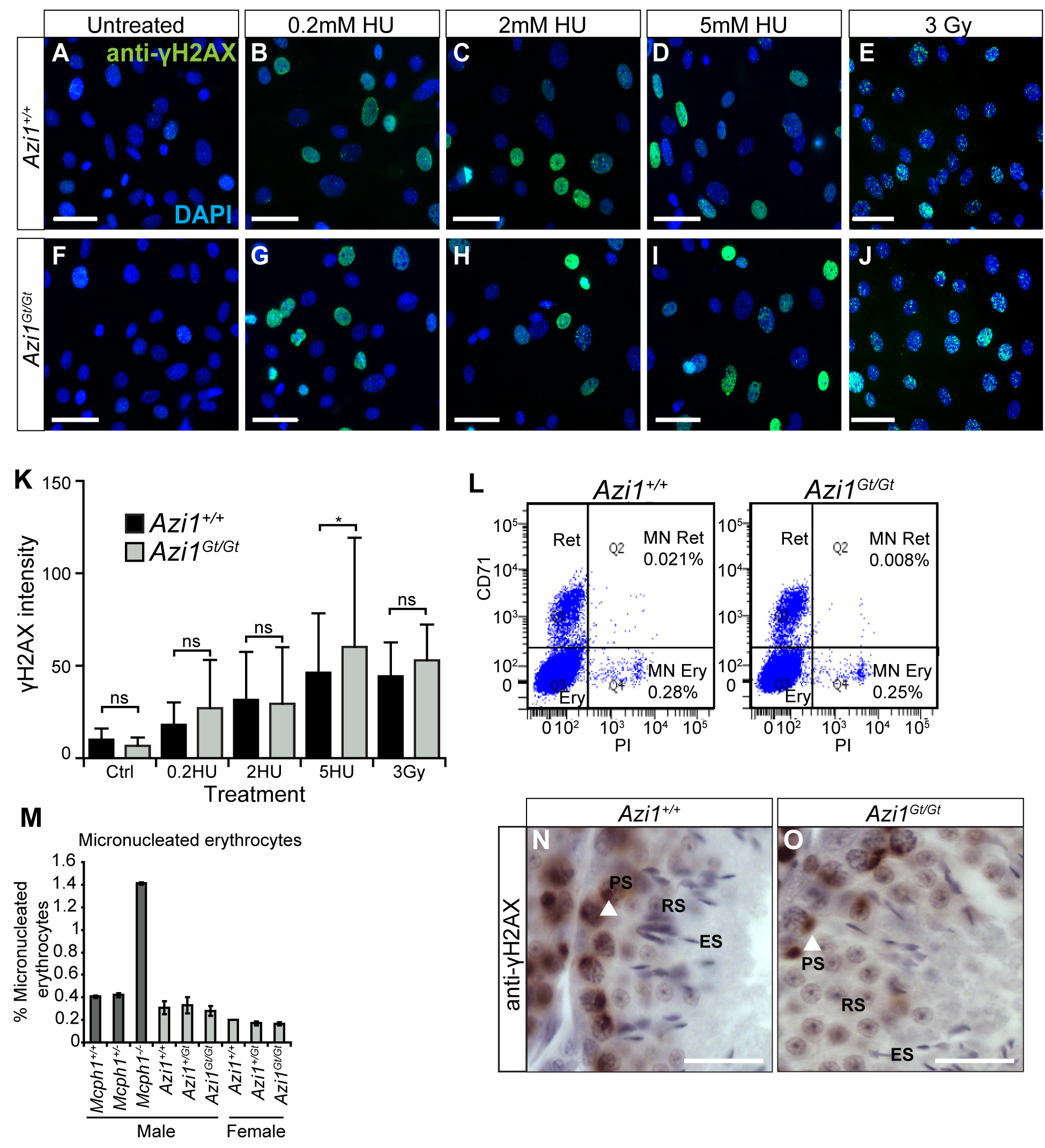

Supplement: Figure S4 — Azi1Gt/Gt mutant mice show no gross increases in DNA damage. DNA damage, measured by anti-γH2AX staining, was assessed in Azi1+/+ and Azi1Gt/Gt MEFs without challenge (A and F), challenged with hydroxyurea (HU) (B–D and G–I) or challenged with 3 Gray (Gy) of ionising radiation (I,J). (K) The intensity of γH2AX staining is plotted in arbitrary units (AU). There is no significant difference in γH2AX staining between Azi1+/+ and Azi1Gt/Gt MEFs when unchallenged, or challenged with up to 2 mM HU or 3 Gy ionising radiation. Azi1Gt/Gt MEFs are more sensitive to high concentrations (5 mM) HU. * P<0.05, ANOVA, n = 3. Shown is mean + STD. (L–M) The percentage of micronuclei in peripheral blood was assessed by flow cytometry. (M) Example FACS data gated to show erythrocytes (Ery, CD71 negative) and reticulocytes (ret, CD71 positive), the high PI containing cells are micronucleated (MN). (N) Plot of the percentage of micronucleated erythrocytes. Mcph1−/− mice show increased numbers of micronucleated erythrocytes, as previously documented (http://www.sanger.ac.uk/mouseportal/phenotyping/MBGX/micronuclei/) (M). Azi1Gt/Gt mice show no change in numbers of micronucleated erythrocytes, suggesting no increased DNA damage in vivo. (N and O) DNA damage was assessed in the testes by anti-γH2AX staining. As DNA double-stranded breaks occur during meiosis, γH2AX staining occurs normally in certain cell types such as in foci in some spermatogonia and preleptotene to zygotene spermatocytes, in the XY body of pachytene spermatocytes and in the nucleus of round spermatids [90]. Comparing stage matched tubules (assessed by serial PAS sections), the Azi1Gt/Gt testes showed no increase in γH2AX staining. In both Azi1+/+ and Azi1Gt/Gt tubules, anti-γH2AX stains the XY body (white arrows) of pachytene spermatocytes (PS) and, less intensely, round spermatids (RS). It is absent from elongating spermatids (ES). Scale bars represent 50 µm (A–J) or 25 µm (N and O). (TIF) [file pgen.1003928.s004.tif]

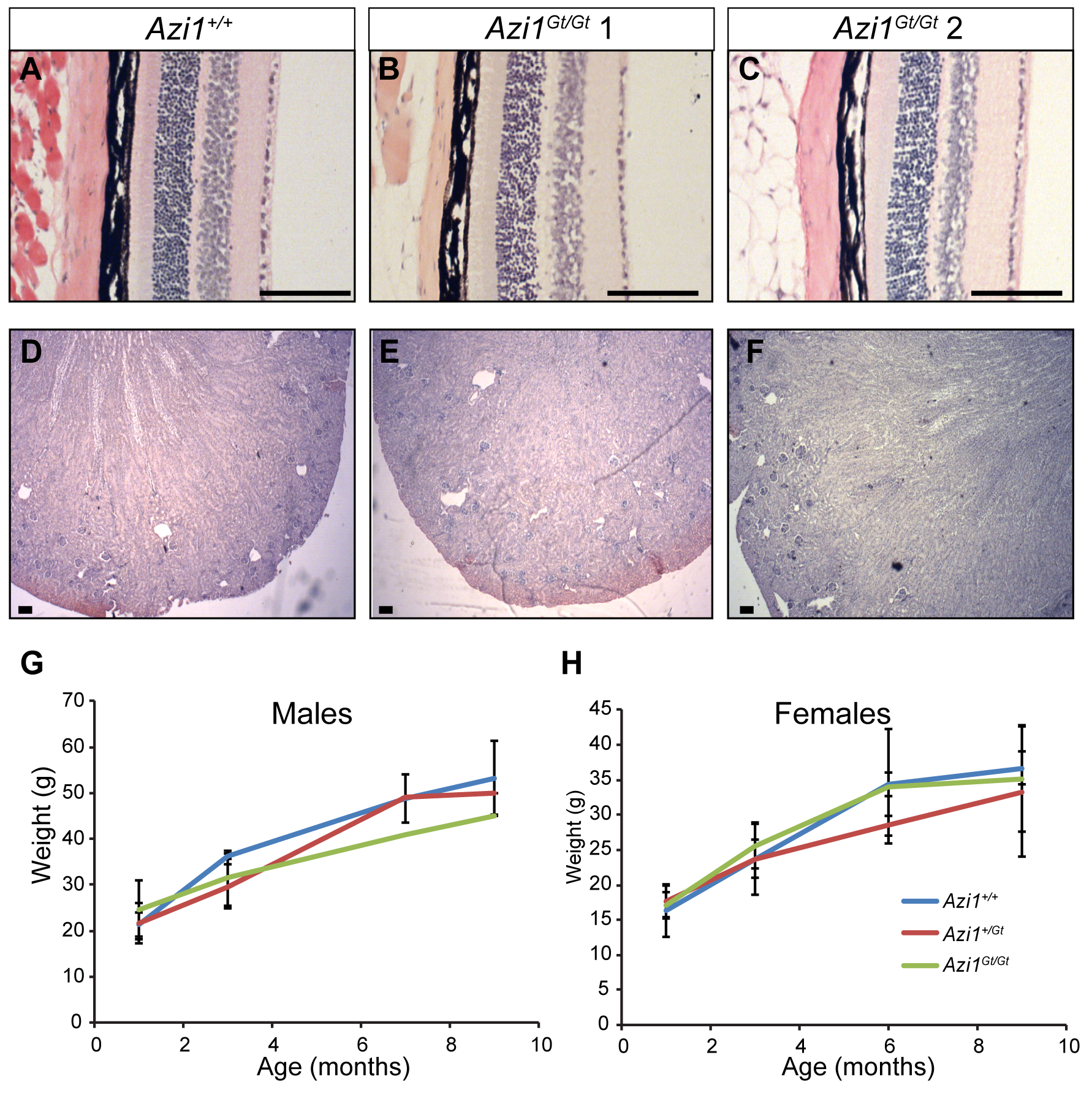

Supplement: Figure S5 — No late-onset ciliopathy phenotypes are observed in Azi1Gt/Gt mice. (A–C) Eyes of seven mutant mice at 6 months of age were directly examined by opthalmoscope and no retinal degeneration was observed (data not shown). This was confirmed histologically on H&E stained wax sections in four mutants. Shown are representative sections of the retina from wild type (A) or two Azi1 null mice (B and C). (D–F) Kidneys were taken from seven mutants aged 6 months or more, and no kidney cysts were seen. This was confirmed by H&E stained sections for four mutants. (E and F) Representative kidney sections from 6 months old Azi1Gt/Gt mice, and Azi1Gt/+ littermate, showing no cysts in the mutant kidneys. (G and H) The weight of male (G) and female (H) Azi1Gt/Gt mice is no different to Azi1+/+ and Azi1Gt/+ mice at 1–9 months old. Scale bars represent 50 µm. (TIF) [file pgen.1003928.s005.tif]

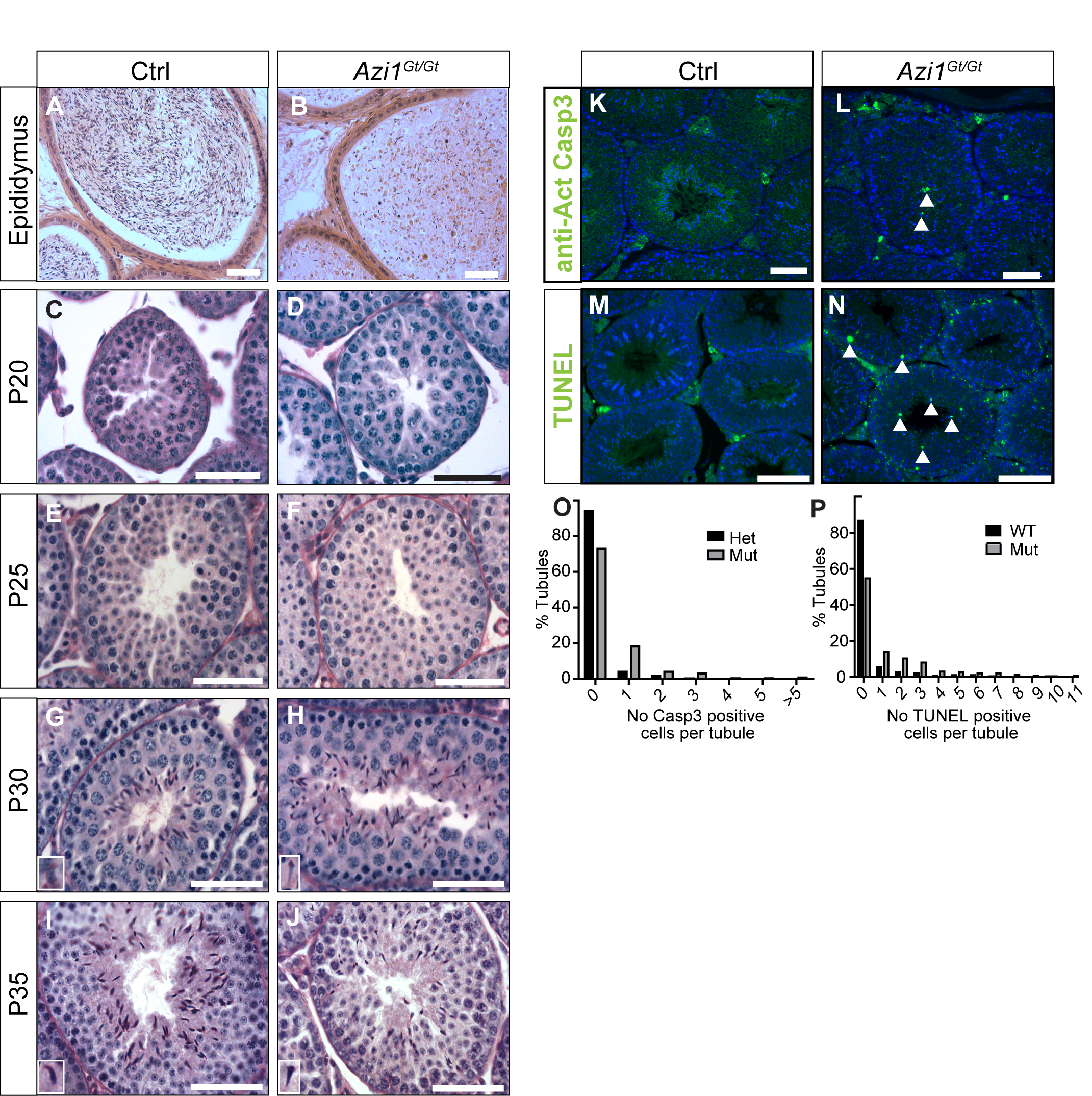

Supplement: Figure S6 — Azi1Gt/Gt sperm morphological defects begin during spermatid elongation, leading to increased apoptosis in adult testes. (A–B) H&E stained sections of adult epididymides, showing a dramatic reduction in the number of sperm present in the Azi1Gt/Gt epididymis. (C–J) PAS stained sections of control (C, E, G and I) or Azi1Gt/Gt (D, F, H and J) tubules from P20 (C and D), P25 (E and F), P30 (G and H) and P35 (I and J) mice, showing the first coordinated wave of spermatogenesis. Azi1Gt/Gt tubules resembled wild type at P20 and P25, suggesting spermatogenesis progresses normally until spermatid elongation. By P30, the abnormalities seen in adult testes (Figure 6C–L) become apparent in the Azi1Gt/Gt tubules, such as a lack of sperm tails (compare lumens in H and J to G and I), disorganisation of the tubules, as demonstrated by the misorientation and mislocalisation of elongating spermatids in H and J, and morphologically abnormal spermatid heads (insets). (K and L) Sections of adult testes anti-activated Caspase 3a (anti-Act Casp3), showing a significant increase in the number of cells undergoing apoptosis in Azi1Gt/Gt tubules (marked by white arrowheads). Quantified in (O) (P<0.0001, n = 3, Mann Whitney U test). (M and N) Adult testes sections labelled with TUNEL, showing an increase in the number of dying cells in Azi1Gt/Gt tubules (marked by white arrowheads), although this is not quite statistically significant. Quantified in (P) (P = 0.051, n = 3, Mann Whitney U test). Scale bars represent 50 µm (A–J) or 100 µm (K–N). (TIF) [file pgen.1003928.s006.tif]

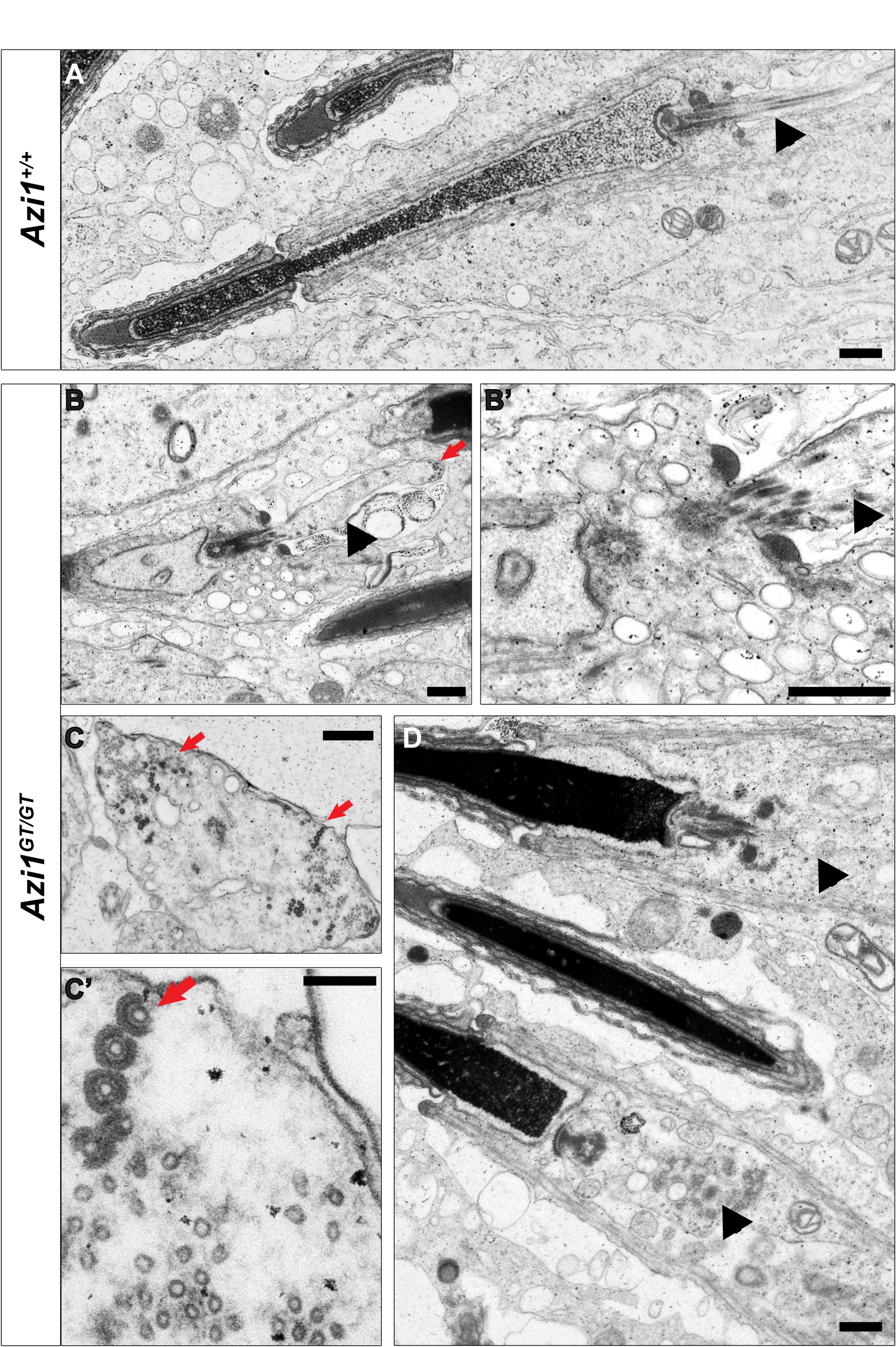

Supplement: Figure S7 — Azi1 null spermatids exhibit IFT-like trafficking defects. (A) In control testes, an elongated spermatid with progressively condensed nucleus with well-defined basal plate where the centriolar complex with extending flagella (arrowhead) has lodged, forming the head-tail connecting apparatus (HTCA). (B, B′) Serial sections of Azi1Gt/Gt elongated testicular spermatid with abnormally swollen and truncated flagellar structure (arrowhead) with no clear axoneme beyond the electron-dense annulus. (B,C,C′) Accumulation of IFT-cargo microtubules visible at distal tip of structure (red arrow), with associated outer dense fibres. C′ is higher magnification view of C, another distal cytoplasmic accumulation of microtubules and outer dense fibres. (D) Absence of discernible flagellar structures associated with HTCAs and annuli is the primary phenotype of Azi1 mutant spermatids. Scale bar represents 500 nm (A, B, B′, C and D) or 100 nm (C′). (TIF) [file pgen.1003928.s007.tif]

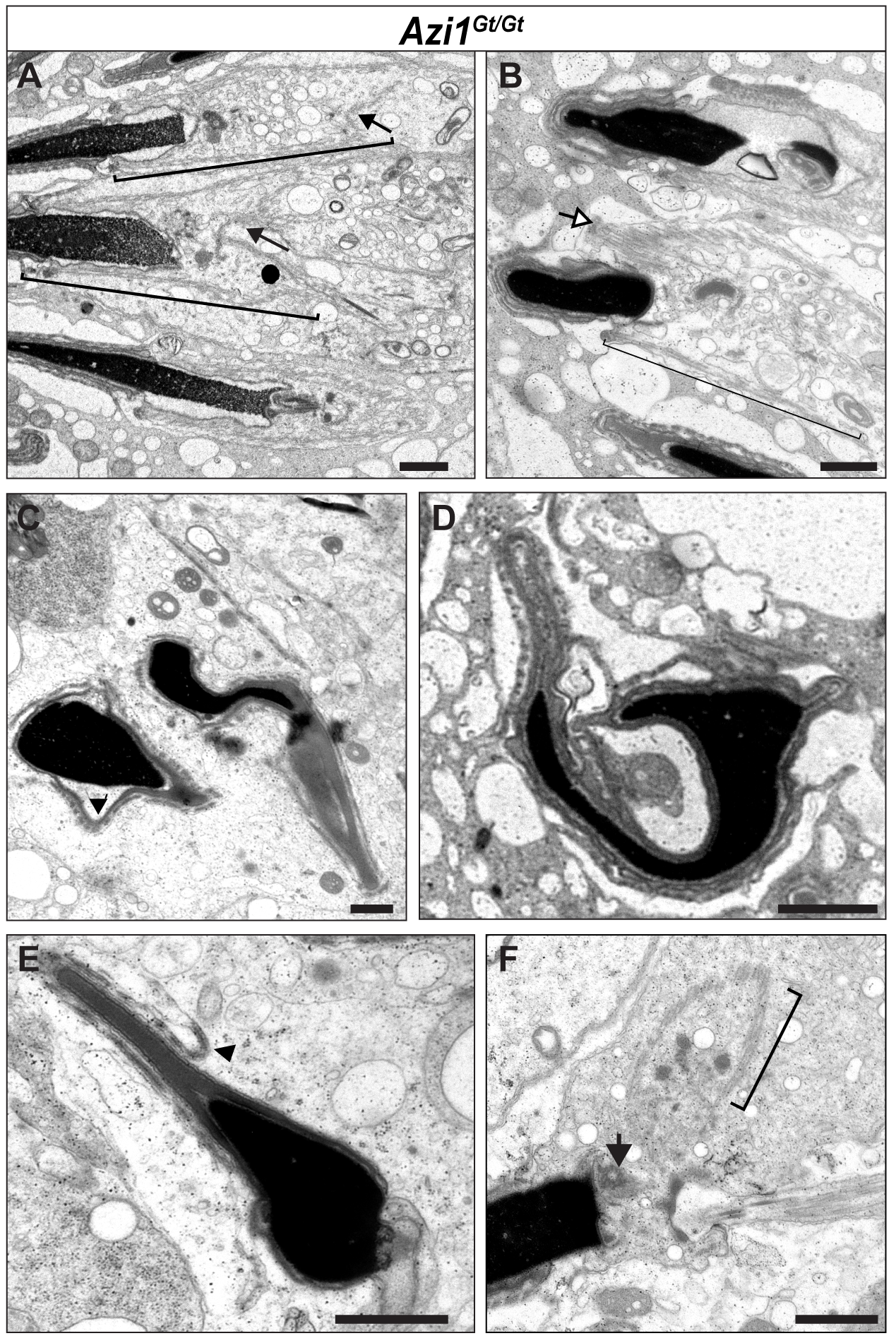

Supplement: Figure S8 — Azi1 mutant spermatids show defects in manchette structure, nuclear abnormalities and HTCA alignment. (A and B) Further examples of Azi1 mutant manchette defects, including kinking (A arrows and brackets) and misnucleation (open arrow) (B). (C–E) Defects in intramanchette transport can lead to abnormalities in nuclear morphology as well as acrosome defects (arrowheads C and E). (F) A further example of a misaligned HTCA, as in Figure 8H, with the implantation fossa off-centre (short arrow). Brackets mark ectopic microtubules, possibly ectopic manchette. (TIF) [file pgen.1003928.s008.tif]
